# Preparation of Tyrian Purple (6,6′-Dibromoindigo): Past and Present

**DOI:** 10.3390/molecules15085473

**Published:** 2010-08-10

**Authors:** Joel L. Wolk, Aryeh A. Frimer

**Affiliations:** Department of Chemistry, Bar-Ilan University, Ramat-Gan 52900, Israel

**Keywords:** 6,6’-dibromoindigo, Tyrian purple, synthesis

## Abstract

Over the past century, various synthetic approaches have been suggested to the most famous dye of antiquity, Tyrian purple (6,6′-dibromoindigo). These synthetic routes have been exhaustively surveyed and critically evaluated from the perspective of convenience, cost, safety and yield.

## 1. Introduction

**Scheme 1:**
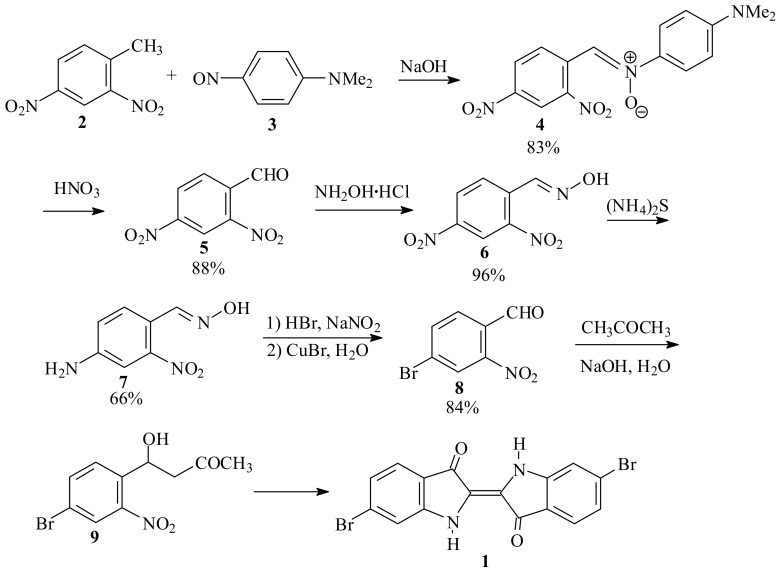
Sachs and Kempf synthesis 6,6′-dibromoindigo (**1**).

6,6′-Dibromoindigo (**1;** see Scheme 1) is the chemical structure of the major component of Tyrian purple, the most famous dye of antiquity [1,2]. From ancient times the dye has been produced from secretions of various species of snails found off the Atlantic and Mediterranean coasts. Due to the minute amounts of dye found in the snails, the dye has always been very costly. Paul Friedländer, who in 1909 first identified the structure of the dye as 6,6′-dibromoindigo, required 12,000 *Murex brandaris* snails to produce 1.4 g of pure pigment [3]. Over the past century a variety of groups have undertaken to develop rational syntheses of this historic dye. Their efforts are surveyed and critically evaluated herein from the perspective of convenience, cost, safety and yield. Several possible improvements are also proposed.

## 2. Preparation Tyrian Purple from 4-Bromo-2-nitrobenzaldehyde

### 2.1. The original synthesis and its elaborations

Nearly all known syntheses of 6,6′-dibromoindigo (**1**) are based on the oxidative coupling of a 6‑bromoindole derivative, which is usually generated *in situ*. Thus, the first synthesis of **1**, reported in 1903 by Sachs and Kempf [4] (Scheme 1), was based on the Claisen condensation of 4-bromo-2-nitrobenzaldehyde (**8**) with acetone, in analogy to the Baeyer-Drewsen process for the manufacture of indigo [5,6]. 

The substituted benzaldehyde **8** was prepared, in five steps starting from 2,4-dinitrotoluene (**2**), in an overall yield of about 34% [4,7,8]. Under the reaction conditions, the hydroxyketone **9** spontaneously cyclizes and undergoes oxidative coupling, but it could be isolated using trisodium phosphate instead of NaOH in the Claisen condensation [8]. Interestingly, the yield of dibromoindigo in the final step was not reported.

**Scheme 2:**
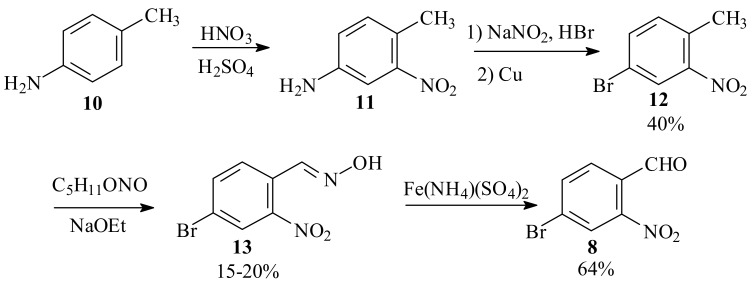
van de Leer approach to bromonitrobenzaldehyde **8**.

This original Sachs and Kempf route is clearly inconvenient, due to the lengthy preparation of the bromonitrobenzaldehyde **8**. Subsequent syntheses were accordingly based on shorter methods for the preparation of this aldehyde. Thus, van der Lee prepared **8** in 4 steps from *p*-toluidine (**10**) [9] (Scheme 2). In the latter procedure, **10** is nitrated in concentrated sulfuric acid to give 4-amino-2-nitrotoluene (**11**) or its sulfate [10,11,12,13,14,15,], which is then diazotized and converted to 4-bromo-2-nitrotoluene (**12**) in a Sandmeyer reaction or one of its variants [9,11,12,16,17,18,19,20,21]. The substituted toluene is then condensed with amyl nitrite [22,23] to give the oxime **13**. The latter is then oxidatively cleaved with ferric ammonium sulfate to give the desired aldehyde **8**.

This synthesis of **2** is indeed shorter, but at the expense of rather low yields. A modest improvement was achieved via a related scheme by Rottig [24], who used ethyl instead of amyl nitrite for the conversion of toluene **12** to oxime **13**. Benzaldehyde **8** was subsequently condensed with acetone in the usual way to give dibromoindigo **1**. However, despite these improvements, the reported overall yield of **1** based on the starting toluidine was only 5.5% [24].

**Scheme 3:**
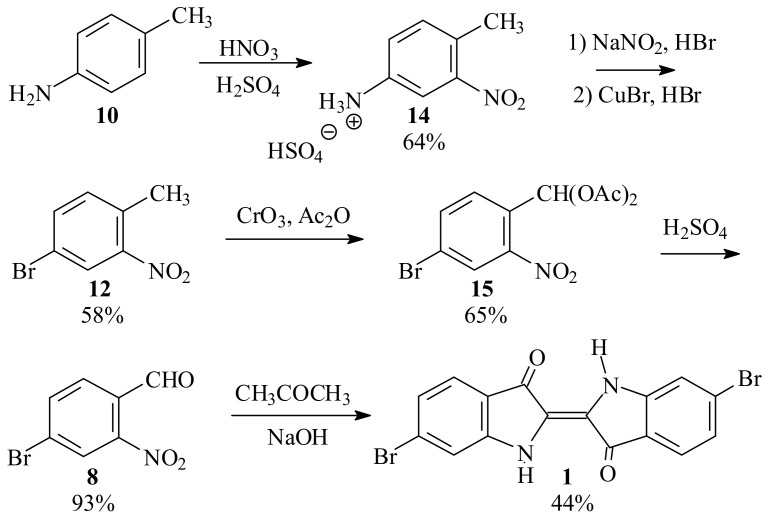
Imming route [33] to Tyrian purple.

An additional drawback of the above scheme is that even the modest yields of the benzaldehyde **8** obtained by reaction of **12** with alkyl nitrites could not be reproduced by later workers. As an alternative to this reaction, Barber and Stickings [25] found that oxidation of **12** with chromic acid in the presence of acetic anhydride was a more reliable method, although the yields were hardly better. The latter method had previously been used to prepare *o*-nitrobenzaldehyde from *o*-nitrotoluene in two steps through the corresponding Diacetate [26,27,28,29]. The chromic acid oxidation of **12** was subsequently used by Pinkney and Chalmers [30] and by Torimoto and coworkers [31] in their syntheses of Tyrian purple, and also by Keinan and coworkers [32]. Recently, the procedure of Pinkney and Chalmers was improved by Imming and coworkers [33], who achieved an overall 10% yield of **1** from **10** (Scheme 3). 

**Scheme 4:**
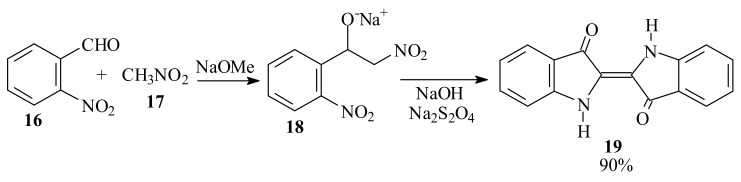
Harley –Mason approach to indigo.

The final condensation of the benzaldehyde **8** with acetone in all these reported syntheses consistently gives yields of no more than about 50% [34]. In 1950, Harley-Mason [35], following the earlier work of Thiele [36], reported that the sodium salt of 2-nitro-1-*o*-nitrophenylethyl alcohol (**18**), obtained by the nitro-aldol condensation (Henry Reaction) of *o*-nitrobenzaldehyde (**16**) with nitromethane (**17**), gives a 90% yield of indigo (**19**) when treated with alkaline sodium dithionite solution (Scheme 4). 

This procedure was later used by Voss and Gerlach [37] and by Cooksey [38], who obtained 66% and 69% yields, respectively, of **1** from the benzaldehyde **8**. However, Imming and coworkers reported [33,39] that, for producing dibromoindigo **1** on a large scale, this procedure was not as practical as the original Baeyer-Drewsen indigo procedure [5,6].

### 2.2. Alternative preparations of 4-bromo-2-nitrotoluene (**12**)

**Scheme 5:**
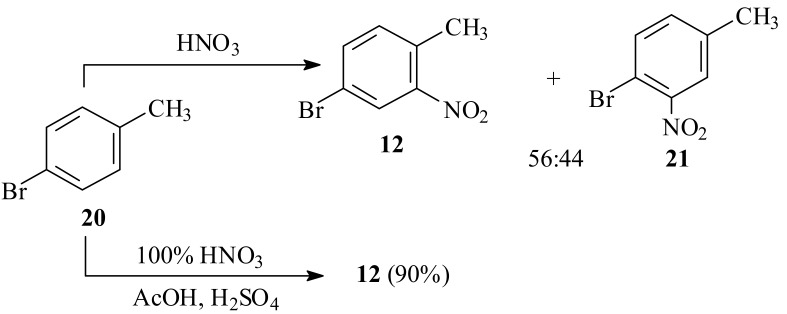
Nitration approach to 4-bromo-2-nitrotoluene (**12**).

All the foregoing syntheses of Tyrian purple rely on 4-bromo-2-nitrobenzaldehyde (**8**) as the key intermediate, which is prepared via 4-bromo-2-nitrotoluene (**12**), which is obtained in turn by diazotization of 4-amino-2-nitrotoluene (**11**) or its sulfate (**14)**. While the Sandmeyer reaction gives good yields, it is rather cumbersome and comparatively expensive, as is the immediate precursor **11**. It is, therefore, reasonable to consider alternative syntheses of **12**; in particular, a one-step preparation from a cheap starting material. The oldest of these consists of nitration of 4-bromotoluene (**20**) [13,40,41,42,43,44,45,46,47,48]. This reaction, however, usually gives a 5:4 mixture of isomer**ic** bromonitrotoluenes **12** and **21**, accompanied by variable amounts of side products, depending on the conditions of the nitration (Scheme 5). Nevertheless, Keinan and coworkers have reported a 90% yield of **12** by performing the nitration with 100% nitric acid in a mixture of acetic acid and 98% sulfuric acid (Scheme 5) [32].

**Scheme 6:**
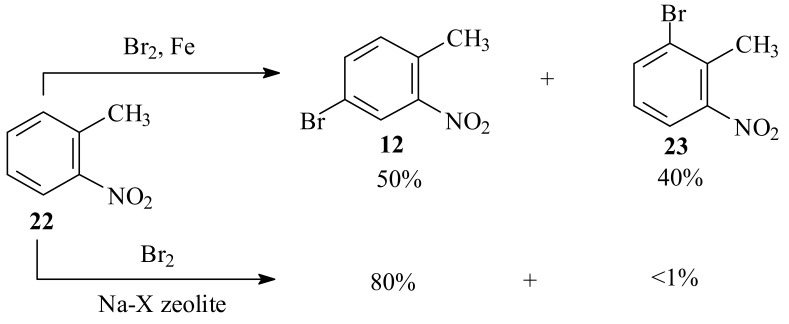
Bromination approach to 4-bromo-2-nitrotoluene (**12**).

A third method for the preparation of **12** consists of brominating *o*-nitrotoluene (**22**) [31,38,49,50,51,52,53,54]. The conventional bromination procedure using catalysis by iron [55] gives here again an isomeric mixture of **12** and 6-bromo-2-nitrotoluene (**23**). Notwithstanding the report by Torimoto and coworkers [31] of a 78% crude yield of **12**, Cooksey [38] confirmed that a 5:4 mixture of **12** and **23** is obtained, from which only a 19% yield of pure **12** could be obtained (by fractional recrystallization from ethanol). More recently, Otake and coworkers [54] have reported that bromination with bromine in the presence of a large amount of a Na-X type zeolite catalyst, without solvent, gives an 80% yield of **12** containing only 0.2% of **23**. When carried out in ethyl acetate as a solvent, the product contained a 99:1 ratio of **12** to **23** (Scheme 6).

### 2.3. Alternative preparations of 4-bromo-2-nitrobenzaldehyde (**8**)

As we have noted, conversion of the bromonitrotoluene **12** to the corresponding benzaldehyde **8** is plagued with inconvenient reactions or low yields. In general the oxidation of toluenes to benzaldehydes is an important industrial process for which a universally optimal procedure has not yet been found. Regarding our case, we note that in their original synthesis of Tyrian purple (Scheme 1), Sachs and Kempf [7] reported a 70% two-step conversion of 2,4-dintrotoluene (**2**) to 2,4-dinitro-benzaldehyde (**5**), by condensing *p*-nitrosodimethylaniline (**3**), followed by acidic hydrolysis of the intermediate nitrone **4** (substantially lower yields were later reported in an *Organic Synthesis* procedure based on this method [56]). The Sachs procedure is amenable only to the oxidation of toluenes substituted with at least two strongly electron-withdrawing groups in the *ortho-* and *para-* positions [57]. In a variation of this procedure, Barrow and coworkers [58,59] succeeded in preparing a number of aldehydes from the corresponding benzyl halides and aromatic nitroso compounds such as **3**. Nevertheless, Barber and Stickings [25] were unable to prepare **8** by reaction of **12** with bromine and **3**. 

**Scheme 7:**
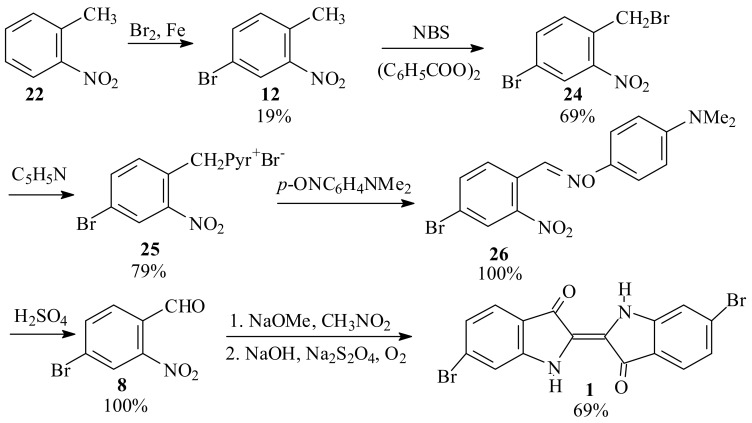
Kröhnke conversion of 4-bromo-2-nitrotolune (**8**) to benzaldehyde (**12**).

However, a more versatile procedure was developed by Kröhnke and coworkers [60,61,62,63,64], who found that conversion of the alkyl bromide group first to its pyridinium salt facilitates subsequent reaction with the aromatic nitroso compound to give the nitrone. An application of the Kröhnke method by Clarke [65] gave several substituted *o*-nitrobenzaldehydes in high overall yields from the respective toluenes. This application has served for a *Organic Synthesis* procedure by Kalir [66] for the preparation of *o*-nitrobenzaldehyde (**16**) in a 47-53% overall yield, and as the basis of a synthesis of **8** by Danieli and coworkers [67]. Cooksey [38] likewise reported a 55% overall yield of **8** using the Kröhnke procedure starting from **12** as part of his complete synthesis of **1** (Scheme 7). Although the Kröhnke procedure involves four steps for converting the substituted toluene to the respective benzaldehyde, it is relatively convenient. Indeed, the only step with a long reaction time is the benzylic bromination of the bromonitrotoluene **12**.

**Scheme 8:**
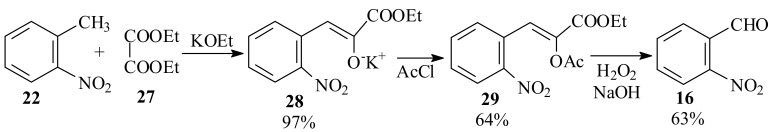
Transformation of o-nitrotoluenes to o-nitrobenzaldehydes.

Two additional methods of converting *o*-nitro-substituted toluenes to the corresponding benzaldehydes are also worth mentioning, although they have not been specifically applied to the synthesis of **8**. These methods start with the first steps of the Reissert [68,69] and Batcho-Leimgruber [70,71,72,73] indole syntheses, respectively. In the first method, the toluene is condensed with diethyl oxalate (**27**), and the enolate anion of the resulting phenylpyruvate ester (**28**) is acetylated and oxidized [74] (Scheme 8). The potassium ethoxide solution needed for the condensation can be prepared by treating an alcoholic potassium hydroxide solution with calcium oxide, thus obviating the need for using metallic potassium [75]. 

**Scheme 9:**
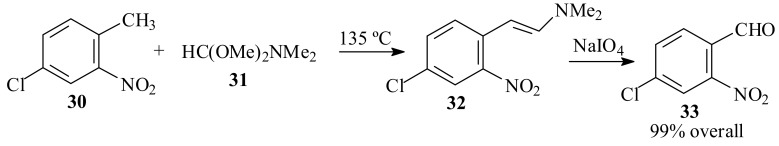
Alternative transformation of o-nitrotoluenes to o-nitrobenzaldehydes.

In the second method, the toluene is condensed with dimethylformamide dimethyl acetal (**31**) and the resulting β-aminostyrene is then oxidized, either catalytically with oxygen [76] or stoichiometrically with sodium periodate [77]. The latter procedure was recently reported to give a nearly quantitative yield of 4-chloro-2-nitrobenzaldehyde (**33**) from 4-chloro-2-nitrotoluene (**30**) [78] (Scheme 9). Both of the indole syntheses mentioned above have been applied to the preparation of 6-bromoindole, as seen below.

**Scheme 10:**
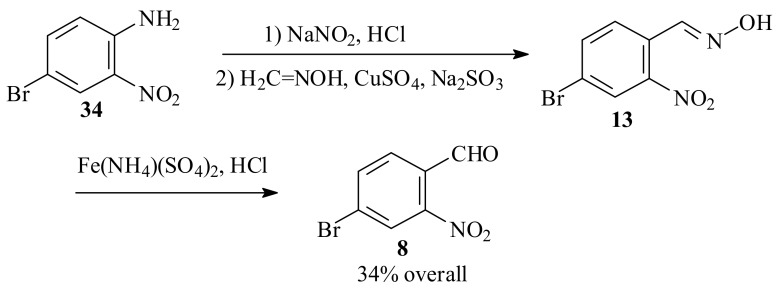
Beech method for the synthesis of 4-bromo-2-nitrobenzaldehyde (**8**).

A completely different approach to substituted benzaldehydes, which does not proceed from the corresponding toluenes, has been developed by Beech [79]. In this synthesis, a diazotized aniline is treated with a solution of formaldoxime in the presence of copper sulfate and sodium sulfite. The intermediate benzaldoxime is then cleaved with ferric ammonium sulfate to give the free benzaldehyde. The Beech method has been applied to the synthesis of a number of substituted benzaldehydes [79,80], including those containing an *ortho* nitro group [81,82,83,]. In particular, it was used by Dandegaonker [84] to prepare **8** in 34% yield from 4-bromo-2-nitroaniline (**34**) (Scheme 10).

**Scheme 11:**
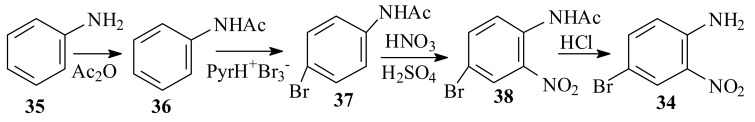
Synthesis of bromonitroaniline **34**.

The starting bromonitroaniline **34** has been prepared by a number of methods, but the most popular are 1) nitration of *p*-bromoaniline or its derivatives [50,85,86,87,88,89,90,91,92,93,94], and 2) bromination of *o*-nitroaniline or its derivatives, usually with bromine in acetic acid [95,96,97,98,99,100,101,102,103,104,105,106,107]. Here, too, each of these methods has its shortcomings. Nitration of *p*-bromoacetanilide (**37**) has been reported in some cases to give 4,6-dibromo-2-nitroacetanilide in variable yields [89,101]. In addition, *p*-bromoacetanilide is not cheap and the nitration method amounts essentially to a three step synthesis starting from acetanilide (**36**), or a four step synthesis from aniline (**35**) [93] (Scheme 11). 

**Scheme 12:**
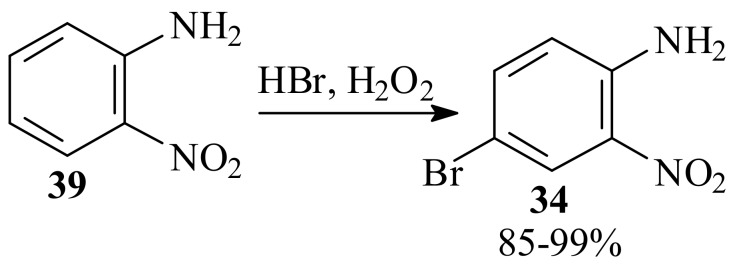
Monobromination of *o*-nitroaniline (**39**).

Bromination of *o*-nitroaniline can likewise lead to formation of the dibrominated product [98, 102,103,104,107], unless less than one equivalent of bromine is used [106]. However, newer, milder synthetic reagents have made the bromination of *o*-nitroaniline (**39**) an attractive route to the required bromonitroaniline **34**. Among these, the most convenient appears to be hydrobromic acid with hydrogen peroxide (Scheme 12) [108,109,110,111]. Other reagents include tetrabromocyclohexadienone [112,113], N-bromosuccinimide [114,115,116], and bromine supported on an ion-exchange resin [117].

**Scheme 13:**
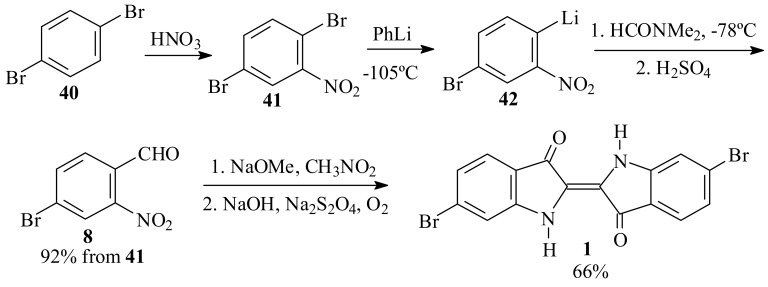
Voss and Gerlach approach to Tyrian purple.

Another novel method for the preparation of **8** has been reported by Voss and Gerlach [37]. In their procedure, p-dibromobenzene is nitrated to give 2,5-dibromonitrobenzene. Lithiation at the 2-position with phenyl- or butyllithium, followed by carbonylation of the lithium derivative with dimethylformamide, furnishes **8** in yields of up to 92% in a one-pot synthesis. Condensation with nitromethane then affords **1** as mentioned above (Scheme 13). This three-step approach to Tyrian purple from *p*-dibromobenzene is indeed very attractive, particularly in light of the high overall yield; nevertheless, subsequent researchers report difficulty with the lithiation step and could not reproduce the published yield [38,118].

## 3. Preparation of Tyrian Purple from 4-Bromo-2-aminobenzoic Acid or Its Derivatives

### 3.1. Friedländer synthesis of Tyrian purple 

**Scheme 14:**
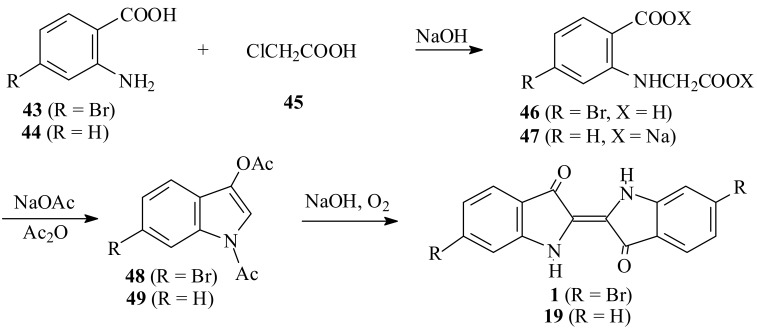
The Friedländer approach to Tyrian purple.

In their original studies on Tyrian purple and related compounds, Friedländer and coworkers [3,11,12] reported a new synthesis of **1** from 4-bromo-2-aminobenzoic acid (**43**). In this procedure, aminobenzoic acid **43** is treated with chloroacetic acid (**45**) [119] yielding the carboxyphenylglycine derivative **46**, which is cyclized in turn to the corresponding diacetylindoxyl **48.** The latter is subsequently hydrolyzed and oxidized in air to give **1** (Scheme 14).

The Friedländer method closely follows the Bayer process for the production of indigo (**19**) from anthranilic acid (**44**) [120], in which the disodium salt of (2-carboxyphenyl)glycine (**47**) is cyclized to diacetylindoxyl (**49**) in acetic anhydride instead of being subjected to alkali fusion, as in the earlier BASF process [121].

**Scheme 15:**
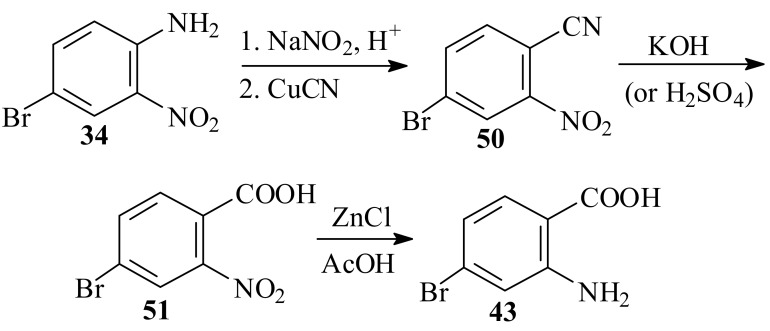
Claus and Scheulen preparation of bromoanthranilic acid **43**.

The major drawback of the Friedländer method is the expense of the starting bromoanthranilic acid **43**. This compound had previously been prepared by Claus and Scheulen [88] from 4-bromo-2-nitroaniline (**34**, from nitration of **39**) by diazotization and conversion to the nitrile **50**, hydrolysis to the acid **51** and reduction with zinc chloride (Scheme 15).

**Scheme 16:**
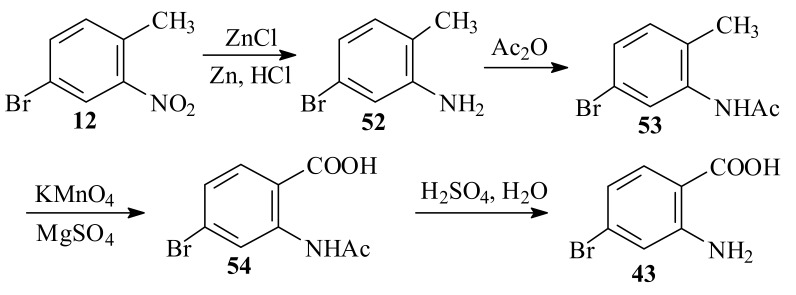
Friedländer approach to bromoanthranilic acid **43**.

Friedländer and coworkers reported two alternative syntheses of **43**. In the first, 4-bromo-2-nitrotoluene (**12**, obtained from **10**) is reduced to the aniline **52**, followed by acetylation, oxidation and hydrolysis (Scheme 16) [3,11,12]. 

**Scheme 17:**
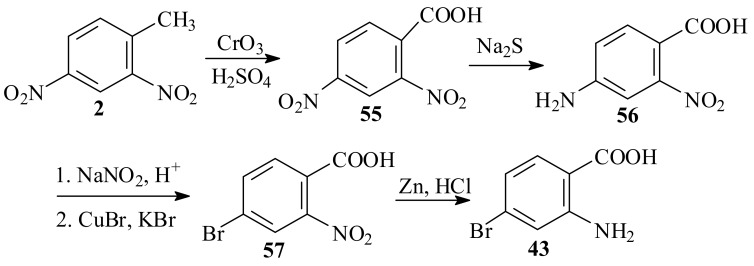
Alternate Friedländer approach to bromoanthranilic acid **43**.

In the second, 2,4-dinitrobenzoic acid (**55**, prepared from 2,4-dinitrotoluene (**2**), presumably by oxidation with chromic acid [122] is partially reduced to 4-amino-2-nitrobenzoic acid (**56**) [123]. The latter is then converted by a Sandmeyer reaction to the bromonitrobenzoic acid **57** and then reduced with zinc and hydrochloric acid, yielding **43** (Scheme 17) [12,124].

The procedures presented above have not been used subsequently to prepare Tyrian purple, but the individual reactions have been used for synthesis of the precursors **43** [50,125,126,127,128,129], **46** [125,129,130,131,132] and **48** [130,131,132].

### 3.2. Alternative syntheses of 4-bromo-2-aminobenzoic acid (**43**) and its derivatives

As noted above, the major drawback to the Friedländer synthesis of **1** is its lengthy preparation of 4-bromo-2-aminobenzoic acid (**43**). For this reason, Imming and coworkers have remarked that this approach is only of “historical interest” [33]. Nevertheless, a shorter route to this compound or its phenylglycine derivative **46** would indeed make it an attractive alternative to the other syntheses reviewed above.

**Scheme 18:**
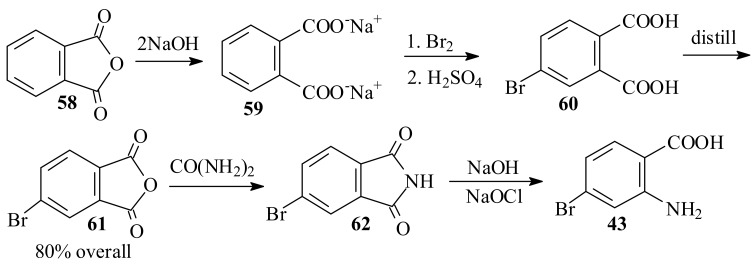
Waldmann procedure to bromoanthranilic acid **43**.

The first alternative to the multistep syntheses of **43** described above was reported by Waldmann (Scheme 18) [133]. 

In this procedure, sodium phthalate (**59**) (derived from phthalic anhydride, **58**) is brominated in aqueous solution to give 4-bromophthalic acid (**60**). The diacid closes to the corresponding anhydride **61** upon distillation, and the latter gives the corresponding imide **62** upon heating with urea. Subsequent Hofmann degradation, analogous to the production of anthranilic acid from phthalimide [134], then gives a product which was identified as 4-bromoanthranilic acid (**43**).

At first glance, the Waldmann procedure seems attractive since the starting material and reagents are all inexpensive and most of the reactions proceed with high yields. Yields of up to 90% of 4-bromophthalic acid (**60**) from bromination of phthalic acid have been reported [135], and conversion of **60** to the anhydride **61** has been reported in nearly quantitative yields [136]. Today **61** is an industrial compound which is readily available in high purity [137,138,139]. 

**Scheme 19:**
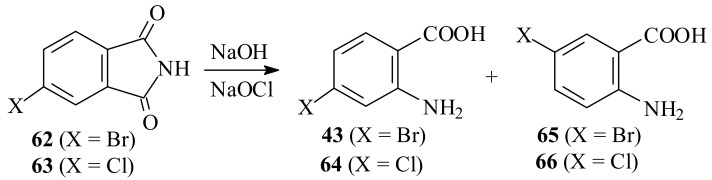
Hofmann reaction of 4-halophthalimides.

The problem with the Waldmann procedure, however, is that the Hofmann reaction of substituted phthalimides is not regioselective. Thus, for example, reaction of 4-chlorophthalimide (**63**) gives a product containing up to 25% of 5-chloroanthranilic acid (**66**), in addition to the 4-substituted main product **64** (Scheme 19) [140]. Although use of benzonitrile as a solvent was reported to give improved selectivity in the synthesis of other substituted anthranilic acids from the corresponding phthalimides, complete selectivity has not been achieved [141]. It is, therefore, to be expected that the Waldmann procedure would give significant amounts of 5-bromoanthranilic acid (**65**) along with the desired acid **43**.

**Scheme 20:**
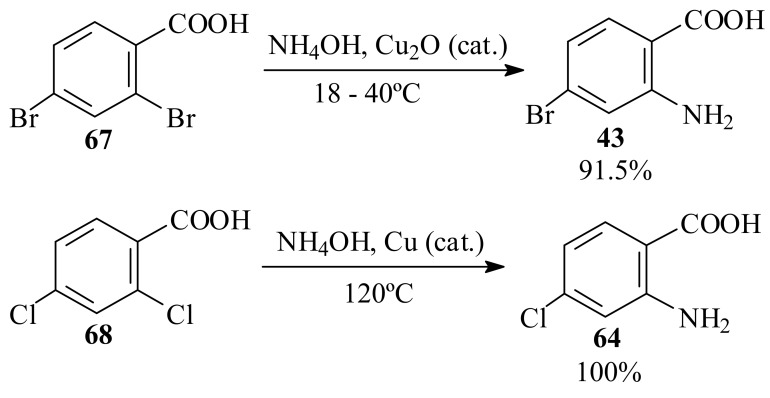
Ullmann condensation of 2,4-dihalobenzoic acid **67** and **68** with ammonia.

An alternative synthesis of 4-bromoanthranilic acid **43** is reported in a recent patent [142] and consists of the Ullmann condensation [143,144] of 2,4-dibromobenzoic acid (**67**) with ammonia catalyzed by cuprous oxide [145] (Scheme 20). This procedure parallels the older synthesis of 4-chloroanthranilic acid (**64**) from 2,4-dichlorobenzoic acid (**68**) and ammonia [146], but as reported [142], requires considerably milder reaction conditions than for the chloro analogue.

**Scheme 21:**
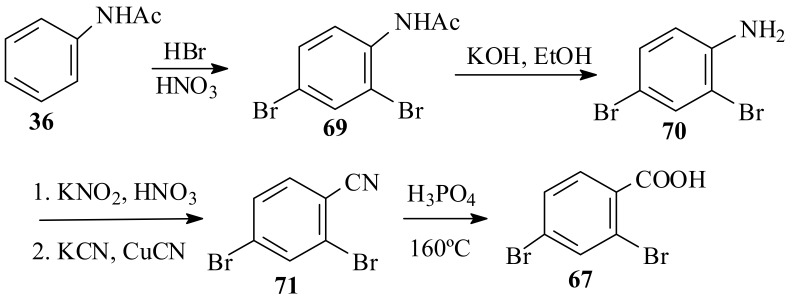
Preparation of 2,4-dibromobenzoic acid (**67**).

The 2,4-dibromobenzoic acid (**67**) required in this procedure is usually prepared via the hydrolysis of 2,4-dibromobenzonitrile (**71**) [147,148,149,150,151,152,153,154,155,156] or by the oxidation of 2,4-dibromotoluene (**45**) [157,158,159,160,161,162,163,164,165,166,167]. Unfortunately, neither of these methods is particularly attractive. The nitrile **71** is almost always made from 2,4-dibromoaniline (**70**) by a Sandmeyer reaction [147,148,150,151,152,153,154], which amounts essentially to a lengthy synthesis starting from acetanilide (**36**) [151,152,153] (Scheme 21). An alternative preparation of **71** based on bromination of 4-bromo-2-nitrobenzonitrile (**50**) with calcium bromide [168] does not appear any more efficient, inasmuch as **50** can itself be converted in two steps to 4-bromoanthranilic acid (**43**), as discussed above (Scheme 15).

**Scheme 22:**
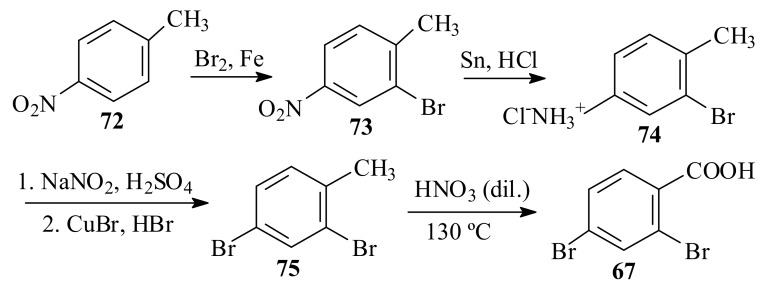
Multi-step approach to 2,4-dibromotoluene (**75**) and 2,4-dibromobenzoic acid (**67**).

**Scheme 23:**
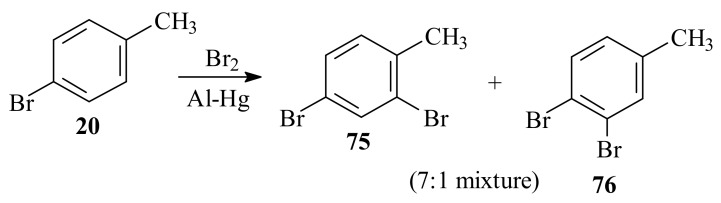
Bromination of p-bromotoluene.

2,4-Dibromotoluene (**75**) is also a potential precursor for 2,4-dibromobenzoic acid (**67**). The former is usually prepared by multistep procedures [157,159,160,161,162,163,164,167,169,170,171], [for example, from 4-nitrotoluene (**72**) [160,164,170] (Scheme 22)], since direct bromination of 4-bromotoluene (**20**) ordinarily gives an 7:1 mixture of **75** and 3,4-dibromotoluene (**76**) [158,160] (Scheme 23).

**Scheme 24:**
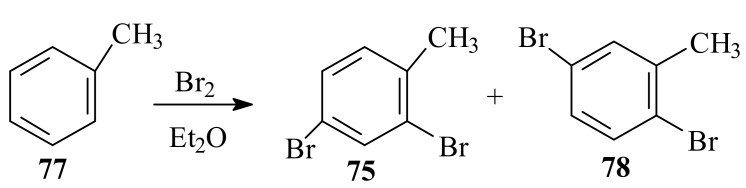
Dibromination of toluene.

Dibromination of toluene (**77**) catalyzed by ethyl ether has likewise been reported to give a mixture of 2,4**-** and 2,5-dibromotoluene (**75** and **78**, respectively) [172] (Scheme 24).

**Scheme 25:**
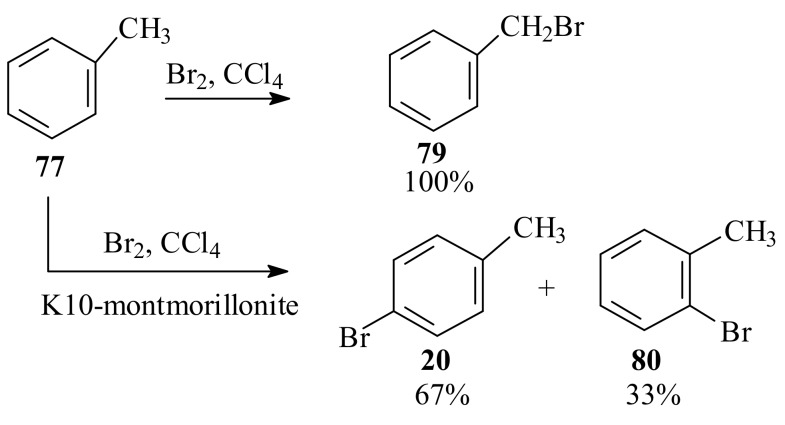
Bromination of toluene.

One patent does report a 70% yield of dibromide **75** from the bromination of toluene (**77**) in carbon tetrachloride in the dark [173]. This is somewhat surprising, however, since bromination of **77** under similar conditions leads to 100% benzylic bromination, although the addition of K10-montmorillonite clay suppresses benzylic bromination yielding a 2:1 mixture of 4- and 2-bromotoluene (**20** and **80**, respectively) [174] (Scheme 25). 

**Scheme 26:**
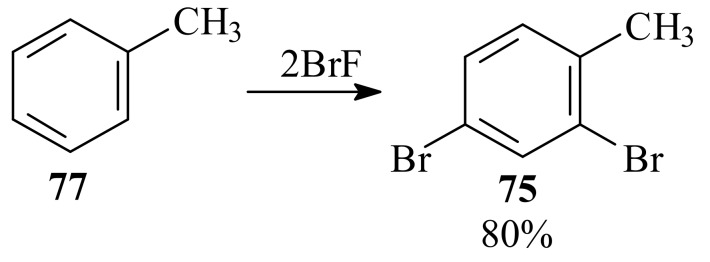
Bromine fluoride bromination of toluene.

The most efficient method reported for production of **75**, to our knowledge, uses bromine fluoride [175,176]. Dibromination of **77** with this reagent gave a reported 80% yield of the desired product [175] (Scheme 26). However, the hazards and cost of the elemental fluorine needed in preparing the reagent make this method rather unappealing. 

**Scheme 27:**
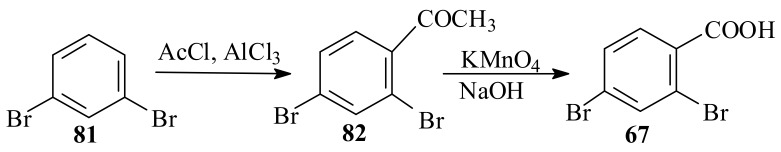
Acetylation of *m*-dibromobenzene.

**Scheme 28:**
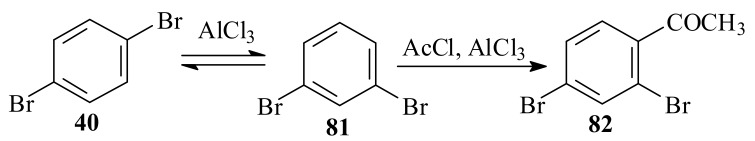
Isomerization/acetylation of *p*-dibromobenzene.

A more promising route to 2,4-dibromobenzoic acid consists of the oxidation of 2′,4′-dibromoacetophenone (**82**) [142,177,178]. The latter compound is readily available through Friedel-Crafts acetylation of 1,3-dibromobenzene (**81**)[177,179,180] (Scheme 27), as well as of the less expensive 1,4-dibromobenzene (**40**) (Scheme 28). 

Not unexpectedly, there are also literature reports indicating that the products of the acetylation of 1,4-dibromobenzene (**40**) are 4′-bromoacetophenone [181] and 2′,5′-dibromoacetophenone [177,182,183,184,185]. However, that the main product is the desired **82** is plausible, given that **40** is rapidly transformed in the presence of the free Lewis acid catalyst to a mixture of isomers in which **81** predominates [186,187,188,189,]. This isomerization reaction is, in fact, the basis for an industrial process for the manufacture of **81** [190,191,192]. Troyanov and Dibinskaya [178] found that no dibromoacetophenone was formed when **40** was treated with a solution of the preformed acetylating complex, AlCl_3_∙CH_3_COCl, without excess aluminum chloride (the Perrier method [194,195]), and thus concluded that the isomerization takes place under the usual reaction conditions in which excess aluminum chloride is present.

The oxidation of the acetophenone **82** to the benzoic acid **67** has usually been performed by basic permanganate. Other potentially attractive methods for this transformation include the haloform reaction [196,197,198,199,200] and catalytic oxidation with molecular oxygen [201,202,203,204,205]. Catalytic processes developed specifically for oxidation of substituted toluenes with oxygen [166,206,207,208,209,210,211] might also be considered.

**Scheme 29:**
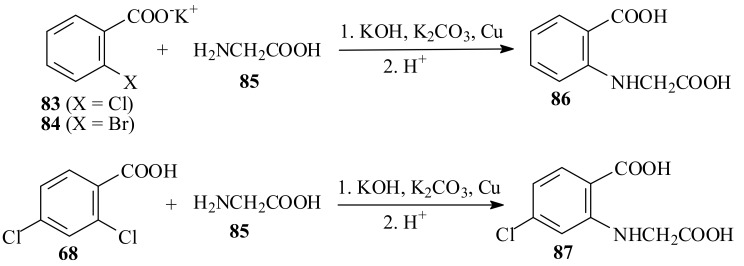
Ullmann condensation preparation of n-(2-carboxyphenyl)glycines **86** and **87**.

The Ullmann condensation, mentioned above (Scheme 20), has also been used to prepare analogues of *N*-(5-bromo-2-carboxyphenyl)glycine (**46**) directly by reaction of *o*-halo substituted benzoic acids with glycine (**85**). Thus, *N*-(2-carboxyphenyl)glycine (**86**) was prepared long ago by condensation of **85** with salts of 2-chlorobenzoic acid (**83**) or 2-bromobenzoic acid (**84**) [212,213,214,215], and *N*-(5-chloro-2-carboxyphenyl)glycine (**87**) has been reported recently as the condensation product of **85** with of 2,4-dichlorobenzoic acid (**68**) [216,217,218] (Scheme 29). No specific example for the corresponding reaction of 2,4-dibromobenzoic acid (**67**) with glycine is reported, but condensations of **67** with phenoxide [150] and with aniline [219] are known. 

**Scheme 30:**
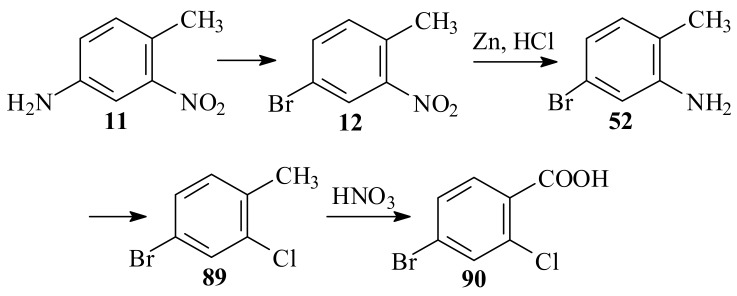
Synthesis of 4-bromo-2-chlorobenzoic acid (**90**) from 4-amino-2-nitrotoluene (**11)**.

**Scheme 31:**
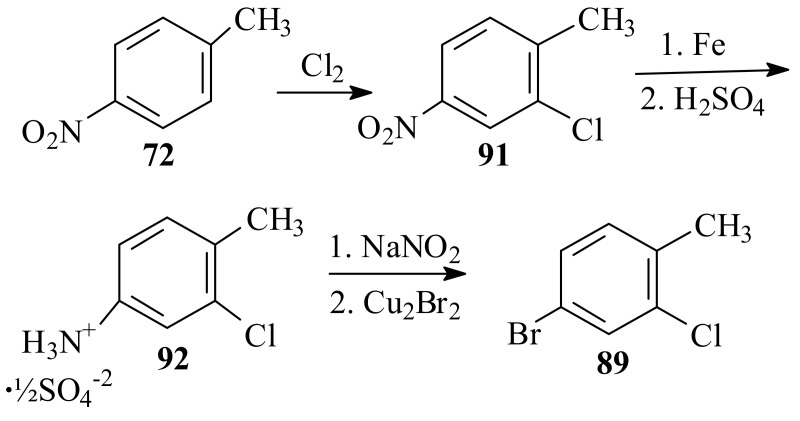
Synthesis of 4-bromo-2-chlorotoluene (**89**) from 4-nitrotoluene (**72)**.

Condensations of 4-bromo-2-chlorobenzoic acid (**90**) with aniline [220] and with *p*-anisidine [221] have likewise been reported to give products in which the *ortho* chlorine atom is replaced by the amino nucleophile. All known preparative syntheses of acid **90** are based on the oxidation of 4-bromo-2-chlorotoluene (**89**) [221,222,223,224]. This, in turn, has been made by multistep procedures, either from 4-amino-2-nitrotoluene (**11)** (Scheme 30) [222,223] or from *p*-nitrotoluene (**72**) in a fashion analogous to the synthesis of the dibromotoluene **75** above (Scheme31)[225,226]. 

**Scheme 32:**
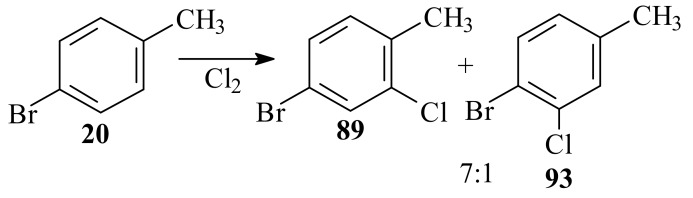
Chlorination of p-bromotoluene.

The chlorination of 4-bromotoluene (**20**), like the bromination, is not completely stereospecific and gives a 7:1 mixture of 4-bromo-2-chlorotoluene (**89)** and the 4-bromo-3-chloro analog (**93**) [223,227] (Scheme 32). 

Judging by the literature reports [220,221] on the condensations of **90**, it seems plausible that condensation of **90** with glycine would furnish the phenylglycine **46**. However, that the *ortho* chlorine ring atom is indeed replaced needs corroboration, inasmuch as recent kinetic studies indicate that the *para* bromine ring atom is more prone to displacement than the *ortho* chlorine [228,229].

## 4. Other Syntheses

### 4.1. From 6,6′-diaminoindigo

**Scheme 33:**
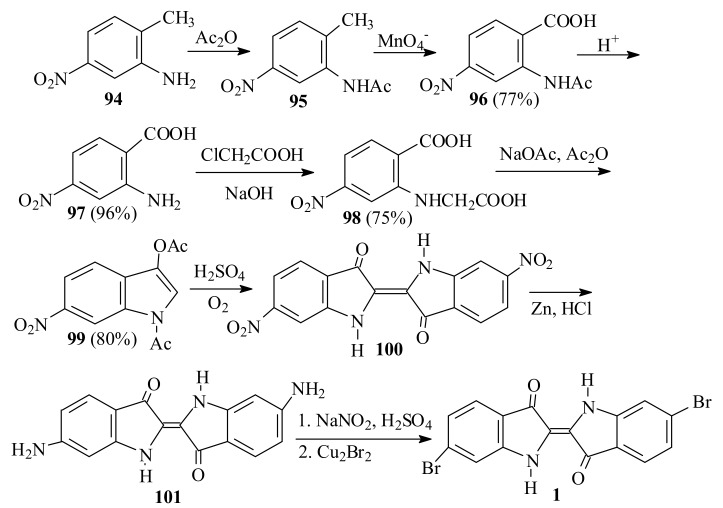
Grandmougin and Seyder synthesis of Tyrian purple.

**Scheme 34:**
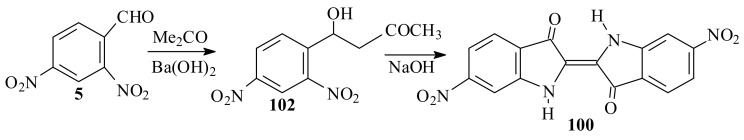
Friedlander preparation of dinitroindigo (**100**).

In 1914 Grandmougin and Seyder reported a synthesis of Tyrian purple by the Sandmeyer reaction of 6,6′-diaminoindigo (**101**) [230]. This is the only known synthesis of **1** in which the bromine atoms are introduced into a previously existing indigo skeleton. The requisite diaminoindigo **101** was prepared by reduction of 6,6′-dinitroindigo (**100**) [231,232] which, in turn, was prepared by way of (2-carboxy-5-nitrophenyl)glycine (**98**) and 1,3-diacetyl-6-nitroindoxyl (**99**), starting from 2-amino-4-nitrotoluene (**94**) [10,233,234,235] (Scheme 33). A shorter alternative synthesis of the intermediate dinitroindigo **100** had previously been reported by Friedländer and Cohn, who prepared it by condensation of 2,4-dinitrobenzaldehyde (**5**) with acetone. [236] (Scheme 34).

**Scheme 35:**
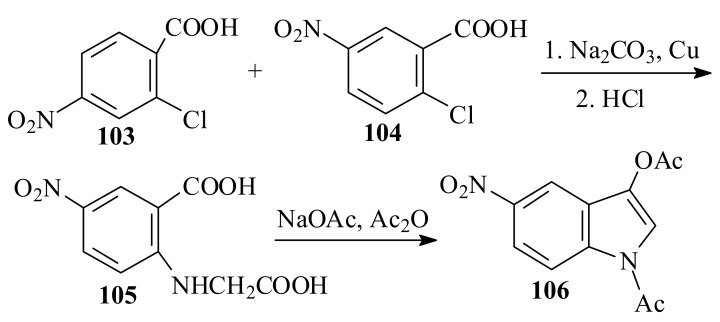
1,3-Diacetyl-5-nitroindoxyl (**106**) from 2-chloro-4-nitrobenzoic acid (**103**).

Whichever way the intermediate dinitroindigo (**100**) is made, this method nevertheless appears rather lengthy. The procedure of Grandmougin and Seyder could possibly be shortened by preparing the phenylglycine **98** by Ullmann condensation of 2-chloro-4-nitrobenzoic acid (**103**) with glycine (**86**). This reaction is not known in the literature; however, the analogous reaction of **86** with the isomeric 2-chloro-5-nitrobenzoic acid (**104**) to give [N-(2-carboxy-4-nitrophenyl)]glycine (**105**) followed by conversion of the latter to 1,3-diacetyl-5-nitroindoxyl (**106**) - is reported [237] (Scheme 35). Even so, the final reaction steps with the sparingly soluble indigo derivatives appear rather cumbersome, and in any case the yield of **1** was not reported.

### 4.2. Dibromoindigo (**1**) from indoles 

**Scheme 36:**
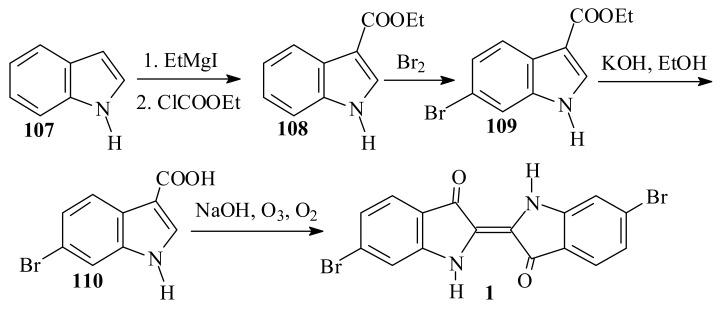
Conversion of indole (**107**) to Tyrian purple.

A novel synthesis of **1,** in which the bromine atom is introduced directly into a previously complete indole ring system, was reported in 1930 by Majima and Kotake [238]. In their synthesis, the Grignard reagent derived from indole [239,240] (**107**) is treated with ethyl chloroformate and the resulting indole-3-carboxylate **108** is brominated. Saponification of the product 6-bromoindole-3-carboxylate (**109**), followed by oxidation with ozonized air, furnished Tyrian purple (Scheme 36).

**Scheme 37:**
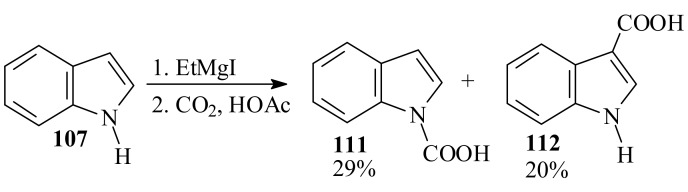
Carboxylation of Indole.

This synthesis would indeed be an attractive route to Tyrian purple (**1**) were it not for the fact that both the Grignard carboxylation and bromination reactions are not regioselective. Thus, both earlier and later reports indicate that only the ring nitrogen atom undergoes metalation [241,242]. In a more recent reinvestigation of the carboxylation of indole (**107**) with carbon dioxide, approximately equal amounts of 1-carboxyindole (**111**) and 3-carboxyindole (**112**) were obtained [243] (Scheme 37). 

**Scheme 38:**
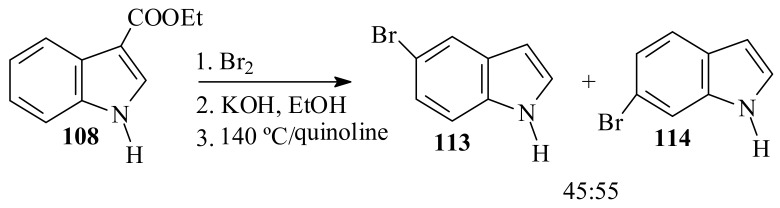
Bromination/decarboxylation of indole **108**.

Moreover, it has also been shown that bromination of **108** followed by decarboxylation gives in fact a 45:55 mixture of 5-bromoindole (**113**) and 6-bromoindole (**114**)[244] (Scheme 38).

### 4.3. Dibromoindigo (**1**) from 6-bromoindole

**Scheme 39:**
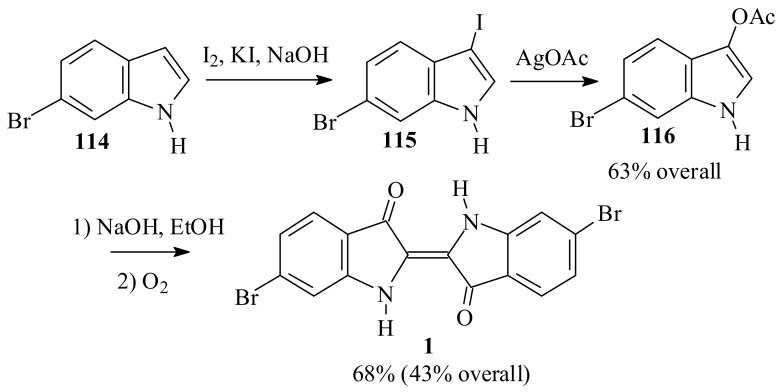
Preparation of Tyrian purple from 6-bromoindole (**114**).

A synthesis of **1** starting from 6-bromoindole (**114**), and based on the biosynthetic pathway of indigo, has recently been published by Tanoue and coworkers [245]. In this procedure, 6-bromoindole (**114**) was iodinated regioselectively, giving 6-bromo-3-iodoindole (**115**). Nucleophilic substitution of the latter with silver acetate gave 3-acetoxy-6-bromoindole (**116**), hydrolysis of which then gave Tyrian purple (**1**) in 43% overall yield (Scheme 39).

**Scheme 40:**
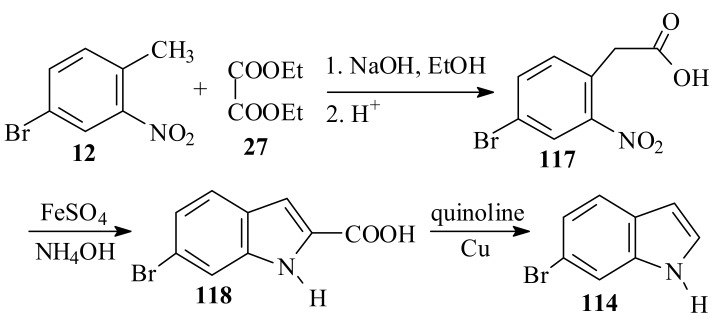
The Reissert indole synthesis.

In terms of simplicity and convenience, this is, indeed, an attractive synthesis. However, the cost of the reagents, particularly the starting material, makes it very expensive route. 6-Bromoindole (**114**) is almost invariably made from 4-bromo-2-nitrotoluene (**12**), either by the Reissert indole synthesis [68,69] or by the Batcho-Leimgruber procedure [70,71,72,73]. In the Reissert synthesis, **12** is condensed with diethyl oxalate (**27**) and the resulting (4-bromo-2-nitrophenyl)pyruvic acid [52,53,54] (**117)** is reduced to give 6-bromo-2-indolecarboxylic acid [52,53] (**118**), which is then decarboxylated [53, 246,247,248,249,250,251,252] (Scheme 40). While both the initial condensation and the final decarboxylation steps have been reported to give good to excellent yields, the reductive cyclization is less efficient and overall yields of the indole **114** are only moderate. Recent enhancements of the Reissert synthesis, such as the use of hydrogenation over platinum and palladium catalysts for the reduction [253], and the use of microwave thermolysis for the decarboxylation [254], have not been applied to the synthesis of **114**. 

**Scheme 41:**
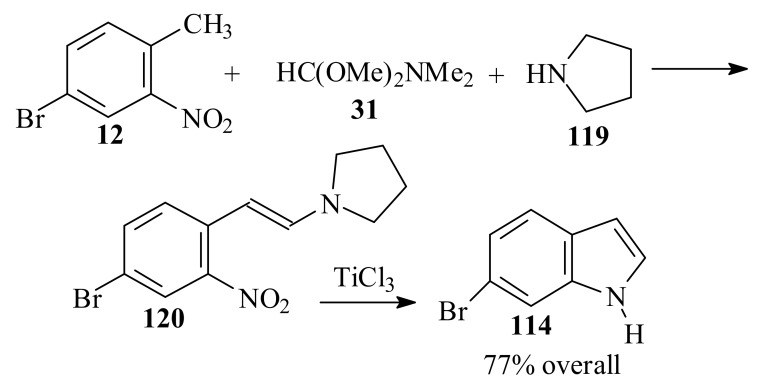
The Batcho-Leimgruber indole synthesis.

The Batcho-Leimgruber synthesis has more recently become the preferred method for preparing **114**. In this procedure, the starting nitrotoluene **12** is condensed with dimethylformamide dimethyl acetal (**31**), usually with an added equivalent of pyrrolidine (**88**) (or with tripiperidinomethane alone [21]), to give the substituted aminostyrene **89**. This is then reduced, affording the desired 6-bromoindole (**81**) (Scheme 41) [21,255,256,257,258,259,260,261,262,263,264,265,266]. While a variety of reducing agents have been used for the second step, buffered aqueous titanous chloride appears to be the most efficient, and overall yields of up to 77% have been reported [257,263]. It is recommended that the reduction step be monitored carefully in order to avoid overreduction to unsubstituted indole, which can make purification of the product difficult [260]. In an alternative application of Batcho-Leimgruber reaction for the synthesis of **81**, 2,4-dinitrotoluene (**3**) is converted to 6-aminoindole by reduction of the respective styrene and subsequently transformed to **81** by a Sandmeyer reaction [267].

**Scheme 42:**
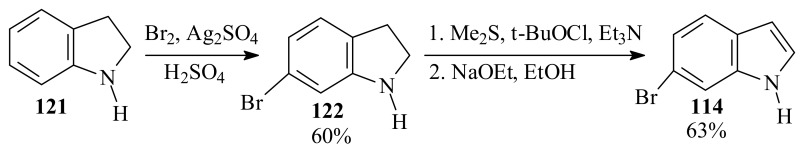
Bromoindole **114** from indoline (**121**).

In an alternative synthesis of **114**, indoline (**121**) is brominated in sulfuric acid in the presence of silver sulfate, giving largely 6-bromoindoline (**122**), accompanied by about 8% of the 4-bromo isomer [268]. The bromoindoline **122** is then dehydrogenated at -65 ºC via the azasulfonium salt with dimethyl sulfide [269], giving **114** (Scheme 42). This scheme enjoys the advantages of an inexpensive starting material and a short reaction sequence. However, the low temperatures needed for the dehydrogenation, plus the need for a chromatographic separation in order to obtain pure product, makes this method rather unsuited for the production of large quantities of 6-bromoindole (**114**).

As noted above, the cost of the starting 6-bromoindole (**114**) and the reagents is the major drawback to the procedure of Tanoue and coworkers [245]. Inasmuch as 4-bromo-2-nitrobenzene (**12**) is the starting material for all practical syntheses of **81**, it would appear more efficient to convert **12** to 4-bromo-2-nitrobenzaldehyde (**8**) and thence directly to **1,** as detailed above, rather than to the indole **114**. We have also noted that the intermediates in the two indole syntheses used for making **114** (*i.e*. the enolate ester of **117** in the Reissert synthesis, and **120** in the Batcho-Leimgruber synthesis) can presumably be oxidized directly to **8** (Scheme 8 and Scheme 9).

**Scheme 43:**
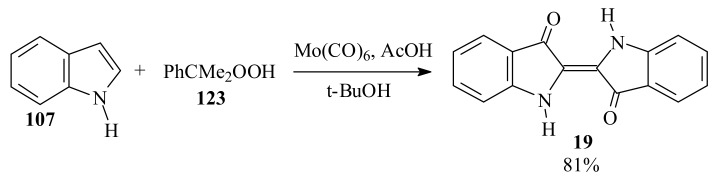
One pot conversion of indole (**107**) to indigo (**119**).

Another alternative to the procedure of Tanoue and coworkers [245], which does use 6-bromoindole (**114**) as a starting material, might possibly be found in a recent one-pot synthesis of indigo from indole (**107**) which uses an organic hydroperoxide and catalysis by a molybdenum complex [270]. In this procedure, yields of up to 81% of indigo (**19**) have been reported when performing the oxidation with cumene hydroperoxide in *t*-butanol and molybdenum hexacarbonyl as a catalyst (Scheme 43). To our knowledge, this procedure has not yet been applied to the synthesis of substituted indigos such as Tyrian purple (**1**).

## 5. Recent Convenient Low-Cost Synthesis of Tyrian Purple

**Scheme 48:**
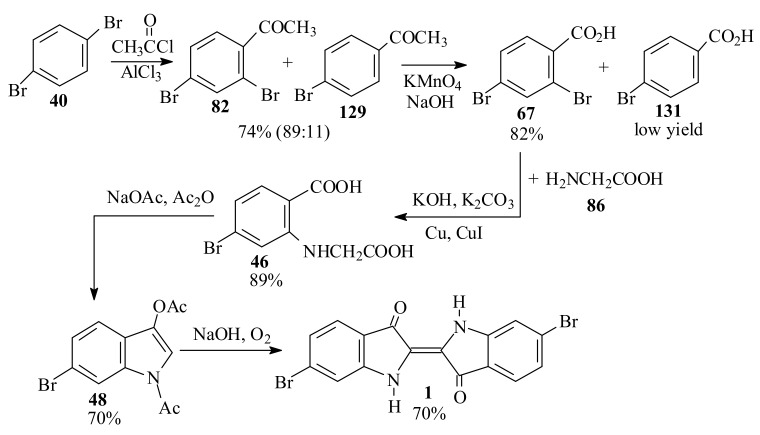
Wolk-Frimer synthetic scheme for the preparation of dibromoindigo (**1**).

Wolk and Frimer [271], have recently reported a five step synthesis of Tyrian purple (**1**), starting from *p*-dibromobenzene (**40;** Scheme 48). The reactions are simple, low cost, safe, high yield procedures. The first step involves the Friedel-Crafts acetylation of *p*-dibromobenzene (**40**) producing 2′,4′-dibromoacetophenone (**82**) as reported by Troyanov and Dibinskaya [178]. In the second step, oxidation of 2′,4′-dibromoacetophenone (**82**) to 2,4-dibromobenzoic acid (**67**), is quite straightforward. The alkaline permanganate oxidation is a standard procedure and the workup is simplified by decomposing the precipitate of manganese dioxide [272]. 

This is followed in the third step by an Ullmann condensation of 2,4-dibromobenzoic acid (**67**) with glycine (**86**) to give the bromocarboxyphenylglycine **46,** which is the novel, key reaction in this synthesis. The condensation of **67** was done in an aqueous system using two equivalents of potassium carbonate and a mixture of copper powder and cuprous iodide as catalysts [273,274,275]. Under these conditions the reaction was vigorous at 50–60 ºC and led to crude yields of **46** of up to 89%. The fourth step involves the Claisen condensation of **46** to give the bromodiacetylindoxyl **48** in a 70% yield. In the final step hydrolysis and oxidation of diacetylindoxyl **48** gives high yields of Tyrian purple (**1**). 

The overall yield of Tyrian purple in this five step synthesis was about 25% based on the starting *p*-dibromobenzene (**40**), and has yet to be fully optimized. Although this yield is significantly lower than that achieved by Voss and Gerlach for their synthesis starting from the same compound [37], Wolk-Frimer procedure has the advantage of not requiring special techniques such as low temperatures or strictly anhydrous conditions, and is, therefore, amenable for student labs and industrial production of larger quantities.

## References

[1] Cooksey C. (2001). Tyrian purple: 6,6′-Dibromoindigo and related compounds. Molecules.

[2] Cooksey C. (1994). Bibliography of Tyrian purple. Dyes Hist. Archaeol..

[3] Friedländer P. (1909). Über den Farbstoff des antiken Purpurs aus murex brandaris. Ber. Deutsch. Chem. Ges..

[4] Sachs F., Kempf R. (1903). Ueber *p*-Halogen-*o*-Nitrobenzaldehyde. Ber. Deutsch. Chem. Ges..

[5] Baeyer A., Drewsen V. (1882). Darstellung von Indigoblau aus Orthonitrobenzaldehyd. Ber. Deutsch. Chem. Ges..

[6] Badische Anilin- und Sodafabrik (1882). Neuerungen in dem Verfahren zur Darstelling des künstlichen Indigos aus dem Orthonitrobenzaldehyd. German Pat..

[7] Sachs Franz, Kempf R. (1902). Ueber eine neue Darstellungsweise von Nitrobenzaldehyden. Ber. Deutsch. Chem. Ges..

[8] Sachs F., Sichel E. (1904). Ueber *p*-substituirte *o*-Nitrobenzaldehyde. Ber. Deutsch. Chem. Ges..

[9] Van der Lee J. (1926). The nitration of cinnamic acid derivatives. Rec. Trav. Chim. Pays-Bas.

[10] Nölting E., Collin A. (1884). Ueber Nitrirung unter verschiedenen Bedingungen. Ber. Deutsch. Chem. Ges..

[11] Friedländer P. (1909). Zur Kenntniss des Farbstoffes des antiken Purpurs aus Murex brandaris. Monatsh. Chem..

[12] Friedländer P., Bruckner S., Deutsch G. (1912). Über Brom- und Methoxyderivate des Indigos. Justus Liebigs Ann. Chem..

[13] Holleman A.F. (1915). Nitration du p-bromotoluène. Rec. Trav. Chim. Pays-Bas.

[14] Ullman F., Dootson P. (1918). Untersuchungen über Farbstoffe der Anthrachinon-acridone-Reihe. Ber. Deutsch. Chem. Ges..

[15] Fierz-David H.E., Blangey L. (1949). Fundamental Processes of Dye Chemistry.

[16] Beilstein F., Kuhlberg A. (1871). Untersuchungen über Isomerie in der Benzoëreihe. IV. Ueber die Bestimmung des chemischen Ortes in einigen Toluolderivaten. Justus Liebigs Ann. Chem..

[17] Gibson C.S., Johnson J.D. A. (1929). 10-chloro-5:10-dihydrophenarsazin and its derivatives. Part IX. The synthesis of nitromethyldiphenylamine-6′-arsinic acids and their conversion into nitromethyl derivatives of 10-chloro-5:10-dihydrophenarsazine. Constitution of 10-chloro-5:10-dihydrophenarsazine. J. Chem. Soc..

[18] Shaw F.R., Turner E.E. (1932). The quantitative nitration of *p*-chloro- and *p*-bromo-toluene. J. Chem. Soc..

[19] Puigdellivol Llobet P. (1978). Procediemento para la obtención de cetonas s-ciclicas de interés farmacológico. Span. Pat..

[20] Kosuge T., Ishida H., Inaba A., Nukaya H. (1985). Synthesis and some reactions of 6-bromooxindole. Chem. Pharm. Bull..

[21] Rinehart K.L., Kobayashi J., Harbour G.C., Gilmore J., Mascal M., Holt T.G., Shield L.S., Lafargue F. (1987). Eudistomins A-Q, β-carbolines from the antiviral Caribbean tunicate *Eudistoma olivaceum*. J. Am. Chem. Soc..

[22] Farbwerke vorm (1899). Meister Lucius & Brüning. Verfahren zur Darstellung von o- und p-Nitrobenzaldoxim und deren Homologen. German Pat..

[23] Lapworth A. (1901). The form of change in organic compounds, and the function of the α-meta-orientating groups. J. Chem. Soc..

[24] Rottig W. (1935). Über eine einfache Synthese des antiken Purpurs. J. Prakt. Chem.

[25] Barber H.J., Stickings C.E. (1945). Phenanthrene-3:6-diamidine. J. Chem. Soc..

[26] Thiele J., Winter E. (1900). Ueber Oxydationen bei Gegenwart von Eissigsäureanhydrid und Schwefelsäure. Justus Liebigs Ann. Chem..

[27] Farbenfabriken vorm. Friedr. Bayer & Co. (1901). Verfahren zur Darstellung von Acetaten aromatischer Aldehyde. German Pat..

[28] Tsang S.M., Wood E.H., Johnson J.R. (1955). *o*-Nitrobenzaldehyde. Org. Synth. Coll..

[29] Nashimura T. (1963). *o*- and p-Nitrobenzaldiacetate. Org. Synth. Coll..

[30] Pinkney J.M., Chalmers J.A. (1979). Synthesizing Tyrian purple. Educ. Chem..

[31] Torimoto N., Morimoto S., Shingaki T. (1991). Synthesis of Tyrian purple as a teaching material. Kagaku to Kyoiku.

[32] Keinan E., Kumar S., Singh S.P., Ghirlando R., Wachtel E.J. (1992). New discotic liquid crystals having a tricycloquinazoline core. Liquid Crystals.

[33] Imming P., Imhof I., Zentgraf M. (2001). An improved synthetic procedure for 6,6′-dibromoindigo (Tyrian purple). Synth. Commun..

[34] Christophersen C., Wätjen F., Buchardt O., Anthoni U. (1978). A revised structure of tyriverdin: the precursor of Tyrian purple. Tetrahedron.

[35] Harley-Mason J. (1950). A new synthesis of indigo. J. Chem. Soc..

[36] Thiele J. (1899). Condensation des Nitromethans mit aromatischen Aldehyden. Ber. Deutsch. Chem. Ges..

[37] Voss G., Gerlach H. (1989). Regioselektiver Brom/Lithium-Austausch bei 2,5-Dibromo-1-nitrobenzol. – Eine einfache Synthese von 4-Brom-2-nitrobenzaldehyd und 6,6′-Dibromoindigo. Chem. Ber..

[38] Cooksey C.J. (1995). Making Tyrian purple. Dyes Hist. Archaeol..

[39] Imming P. (2000).

[40] Wroblewski E. (1873). Ueber einige Haloïdderivate des Toluols. Justus Liebigs Ann. Chem..

[41] Hübner H., Roos P.F. (1873). Ueber isomere Bromtoluidine. Ber. Deutsch. Chem. Ges..

[42] Lellmann E., Just R. (1891). Ueber einige Derivate des Piperidins. Ber. Deutsch. Chem. Ges..

[43] 43 Price C.C., Sears C.A. (1953). Nitryl chloride as a nitrating agent. J. Am. Chem. Soc..

[44] Moodie R.B., Schofield K., Weston J.B. (1976). Electrophilic aromatic substitution. Part XV. The kinetics, mechanism, and products of nitrodebromination in sulfuric acid. J. Chem. Soc. Perkin Trans. 2.

[45] Clemens A.H., Hartshorn M.P., Richards K.E., Wright G.J. (1977). Nitration studies of 4-substituted toluenes, 5-substituted hemimellitenes and acetoxyprehnitene. Aust. J. Chem..

[46] Bushnell G.W., Fischer A., Henderson G.N., Mahasay S.R. (1986). *ipso*-Nitration. XXVII. The crystal structure and stereochemistry of 3-bromo-6-methyl-6-nitrocyclohexa-2,4-dienyl acetate, 5-bromo-2-methyl-6-nitrocyclohexa-2,4-dienyl acetate, and 3-bromo-6-methyl-6-nitrocyclohexa-2,4-dienyl chloride. Can. J. Chem..

[47] Tomokazu G. (1992). Preparation of 4-halomononitrotoluenes. Japanese Pat..

[48] Suzuki H., Mori T. (1994). Ozone-mediated nitration of chloro- and bromo-benzenes and some methyl derivatives with nitrogen dioxide. High *ortho*-directing trends of the chlorine and bromine substituents. J. Chem. Soc. Perkin Trans. 2.

[49] Gluud W. (1915). Notiz über die Bromierung vom *o*-Nitrotoluol und über das *o*-Nitrobenzyl-pyridiniumchlorid. Ber. Deutsch. Chem. Ges..

[50] Frejka J., Vymetal F. (1935). Dérivés halogens de la novocaïne II (*p*-bromo-*o*-aminobenzoyl-diéthylamino-éthanol). Coll. Czech. Chem. Commun..

[51] Mehta D.R., Ayyar P.R. (1942). Bromination of *o*-nitrotoluene and the steric effect of the bromine atom on the relative yields of the 4- and 6-bromo derivatives. J. Univ. Bombay Sci. Phys. Sci. Math. Biol. Sci. Med..

[52] 52 Barltrop J.A., Taylor D.A.H. (1954). Synthesis of lysergic acid. I. Derivatives of indole. J. Chem. Soc..

[53] 53 Plieninger H. (1955). Die Synthese des 4- und 6-Brom-indols und 4- und 6-Amino-indols sowie einige Umsetzungen dieser Verbindungen. Chem. Ber..

[54] Otake H., Tsutsumi H., Murata M. (1997). Production of benzene derivative. Japanese Pat..

[55] Bunnet J.F., Rauhut M.M. (1963). 2-Bromo-3-methylbenzoic acid. Org. Synth. Coll..

[56] Bennett G.M., Bell E.V. (1943). 2,4-Dintrobenzaldehyde. Org. Synth. Coll..

[57] Bayer O., Müller E. (1954). Methoden zur Herstellung und Umwandlung von Aldehyden. Methoden der Organischen Chemie. Band VII, Teil 1: Sauerstoffverbinungen II.

[58] Barrow F., Griffiths E.D. (1921). Condensation of *p*-nitrobenyl chloride with nitroso-compounds. A new mode of formation of *N*-oximino-ethers. J. Chem. Soc..

[59] Barrow F., Griffiths E.D., Bloom E. (1922). *N*-Oximino-ethers. Part II. *N*-Aryl ethers of 2:4- and 2:6-dinitrobenzaldoximes. J. Chem. Soc..

[60] Kröhnke F., Börner E. (1936). Über α-Keto-aldonitrone und eine neue Darstellungsweise von α-Keto-aldehyden. Ber. Deutsch. Chem. Ges..

[61] Kröhnke F. (1938). Über Nitrone. II. Mitteil. Ber. Deutsch. Chem. Ges..

[62] Kröhnke F. (1938). Über Nitrone. III. Mitteil.: *cis*-*trans*-Isomerie bei Anilen?. Ber. Deutsch. Chem. Ges..

[63] Kröhnke F. (1953). Synthesen mit Hilfe von Pyridiniumsalzen. Angew. Chem..

[64] Kröhnke F. (1963). Neuere Methoden der präparativen organischen Chemie. Synthesen mit Hilfe von Pyridiniumsalzen (IV). Angew. Chem..

[65] Clarke K. (1957). Some aromatic aldehydes. J. Chem. Soc..

[66] Kalir A. (1973). *o*-Nitrobenzaldehyde. Org. Synth. Coll..

[67] Danieli R., Maccagnani G., Taddei F. (1968). Ricerche sugli isatogeni. Nota III: Sintesi, iduzione e spettri P.M.R. do esteri isatogenici. Boll. Sci. Fac. Chim. Ind. Bologna.

[68] Reissert A. (1897). Einwirkung von Oxalester und Natriumäthylat auf Nitrotoluole. Synthese nitrirter Phenybrenztarubensäuren. Ber. Deutsch. Chem. Ges..

[69] Noland W.E., Baude F.J. (1973). Ethyl indole-2-carboxylate. Org. Synth. Coll..

[70] Batcho A.D., Leimgruber W. (1973). Process and intermediates for the preparation of indoles from ortho-nitrotoluenes. US Pat.

[71] Batcho A.D., Leimgruber W. (1976). Intermediates for indoles. US Pat..

[72] Batcho A.D., Leimgruber W. (1990). Indoles from 2-methylnitrobenzenes by condensation with formamide acetals followed by reduction: 4-Benzyloxyindole. Org. Synth. Coll..

[73] Clark R.D., Repke D.B. (1984). The Leimgruber-Batcho indole synthesis. Heterocycles.

[74] Marti H.-R., Gnehm R. (1984). Process for producing *o*-nitrobenzaldehyde. US Pat..

[75] Zhang B., Li B., Li A. (1995). Synthesis of potassium enolates of ethyl *o*-nitrophenylpyruvate and the preparation of potassium ethoxide. Huaxue Tongbao.

[76] Blank H.U., Kraus H. (1992). Process for the preparation of benzaldehydes. US Pat..

[77] Vetelino M.G. (1994). A mild method for the conversion of activated aryl methyl groups to carboxaldehydes via the uncatalyzed periodate cleavage of enamines. Tetrahedron Lett..

[78] Caron S., Vazquez E., Stevens R.W., Nakao K., Koike H., Murata Y. (2003). Efficient synthesis of [6-chloro-2-(4-chlorobenzoyl)-1*H*-indol-3-yl]-acetic acid, a novel COX-2 inhibitor. J. Org. Chem..

[79] Beech W.F. (1954). Preparation of aromatic aldehydes and ketones from diazonium salts. J. Chem. Soc..

[80] Jolad S.D., Rajagopal S. (1973). 2-Bromo-4-methylbenzaldehyde. Org. Synth. Coll..

[81] Woodward R.B., Bader F.E., Bickel H., Frey A.J., Kierstead R.W. (1958). Total synthesis of reserpine. Tetrahedron.

[82] Kralt T., Asma W.J., Haeck H.H., Moed H.D. (1961). Reserpine analogs. II. β-Indolylethylamine derivatives. Rec. Trav. Chim. Pays-Bas.

[83] Gal'bershtam M.A., Budarina Z.N. (1969). Synthesis of *para*-substituted *o*-nitrobenzyl alcohols. Zh. Org. Khim..

[84] Dandegaonker S.D. (1964). Nitrohalobenzaldehydes. J. Karnatak Univ..

[85] Hübner H., Retschy H. (1873). Ueber Amidobenzol und eine einfache Darstellung des Metadiamidobenzols (Schmelzp. 102-103°). Ber. Deutsch. Chem. Ges..

[86] Remmers L. (1874). Ueber einige Derivate bromirter Aniline. Ber. Deutsch. Chem. Ges..

[87] Hübner H. (1881). Ueber Anhydroverbingungen. Justus Liebigs Ann. Chem..

[88] Claus A. (1891). Scheulen, M.E. Zur Kenntniss ser Bromnitrobenzoësäuren. J. Prakt. Chem..

[89] Griffith R.H. (1924). Nitration of *p*-bromoacetanilide. J. Chem. Soc..

[90] Henley R.V., Turner E.E. (1930). The scission of diaryl ethers and related compounds by means of piperidine. Part III. The nitration of 2:4-dibromo-2′:4′-dinitrodiphenyl ether and of 2:4-dibromophenyl *p*-toluenesulphonate and benzoate. The chlorination and bromination of *m*-nitrophenol. J. Chem. Soc..

[91] Curd F.H.S., Davey D.G., Stacey G.J. (1949). Synthetic antimalarials. Part XI. The effect of variation of substituents in 2-chloro-3-β-diethylaminoethylaminoquinoxaline. J. Chem. Soc..

[92] Britton D., Noland W.E. (1962). Benzfuroxans. The crystal and molecular structure of 5-chlorobenzfurazan 1-oxide and 5-bromobenzfurazan 1-oxide. J. Org. Chem..

[93] Elder J.W., Paolillo M.A. (1994). 4-Bromo-2-nitroaniline: a multistep synthesis. J. Chem. Ed..

[94] Dirk S.M., Tour J.M. (2003). Synthesis of nitrile-terminated potential molecular electronic devices. Tetrahedron.

[95] Hübner H. (1877). Mittheilungen aus dem Göttinger Laboratorium. IV. Ber. Deutsch. Chem. Ges..

[96] Fuchs W. (1915). Über Bromierung aromatischer Amine. Monatsh. Chem..

[97] Bradfield A.E., Orton K.J.P., Roberts I.C. (1928). Chloramines as halogenating agents. Iodination by a chloramines and an iodide. J. Chem. Soc..

[98] Erickson J.L.E., Dechary J.M., Pullig T.R. (1952). The bromo-2-nitrobenzoic acids. J. Am. Chem. Soc..

[99] Heppolette R.L., Miller J. (1953). The SN mechanism in aromatic compounds. V. Halogen substituents. J. Am. Chem. Soc..

[100] Badger G.M., Sasse W.F.H. (1957). Phenanthridines. Part I. The synthesis of bromophenanthridines. J. Chem. Soc..

[101] Badger G.M., Clark D.J., Davies W., Farrer K.T.H., Kefford N.P. (1957). Thionaphthencarboxylic acids. J. Chem. Soc..

[102] Dandegaonker S.H., Shastri D. (1965). Brombenzimidazole. Monatsh. Chem..

[103] Dandegaonker S.H. (1965). Halogenation of 2-methylbenzimidazole. J. Indian Chem. Soc..

[104] Samuel E.L. (1972). Nitration and bromination products of 2-trifluoromethylbenzenes and their N.M.R. shift assignments. Aust. J. Chem..

[105] Czuba W., Poradowska H. (1974). An unusual course of amination of 6-bromoquinoxaline. Rocz. Chem..

[106] Wilson J.G., Hunt F.C. (1983). Iminodiacetic acid derivatives of benzimidazole. Synthesis of *N*-(benzimidazol-2-ylmethyl)iminodiacetic acids. Aust. J. Chem..

[107] Zhou Q., Huang H., Chen J. (1988). Synthesis of 3,5-dibromo-o-phenylenediamine. Huaxue Shiji.

[108] Islam A.M., Hassan E.A., Hannout I.B., Mourad M.Y. (1970). Halogenation of substituted anilines with halogen acids and hydrogen peroxide. U.A.R. J. Chem..

[109] Hashizume K., Nagano H., Kakoi H., Tanino H., Okada K., Inoue S. (1985). Model experiments on surugatoxin synthesis. II. Synthesis of ethyl 4-amino-2-(6-bromo-2-oxo-3-indolinyl)-3-oxobutyrate hydrochloride. Yakugaku Zasshi.

[110] Okada K. (2000).

[111] Vyas P.V., Bhatt A.K., Ramachandraiah G., Bedekar A.V. (2003). Environmentally benign chlorination and bromination of aromatic amines, hydrocarbons and naphthols. Tetrahedron Lett..

[112] Fox G.J., Hallas G., Hepworth J.D., Paskins K.N. (1988). *para*-Bromination of aromatic amines: 4-bromo-*N,N*-dimethyl-3-(trifluoromethyl)aniline. Org. Synth. Coll..

[113] Sun Q., Gatto B., Yu C., Liu A., Liu L.F., LaVoie E.J. (1995). Synthesis and evaluation of terbenzimidazoles as topoisomerase I inhibitors. J. Med. Chem..

[114] Xu F., Chen C. (1986). NBS-DMF: a mild, selective brominating agent for aromatic amines. Shanghai Keji Daxue Xuebao.

[115] Xu F., Wang Q. (1986). A mild, selective monobromination of organic amines. Huaxue Shiji.

[116] Hersperger R., Dawson J., Mueller T. (2002). Synthesis of 4-(8-benzo[1,2,5]oxadiazol-5-yl-[1,7]naphthyridone-6-yl)-benzoic acid: a potent and selective phosphodiesterase type 4D inhibitor. Bioorg. Med. Chem. Lett..

[117] Yang N.-F., Liang W.-J., Yang L.-W. (1998). The bromination of active aromatic compounds by bromine supported on iron-exchange resin. Youji Huaxue.

[118] Cooksey C. (2000).

[119] Mauthner J., Suida W. (1888). Über Phenylglycin-ortho-carbonsäure, sowie über die Gewinnung von Glycocoll und seinen Derivaten. Monatsh. Chem..

[120] (1900). Farbenfabriken vorm. Friedr. Bayer & Co. Verfahren zur Darstellung von Diacetylindoxyl und Derivaten. German Pat..

[121] (1891). Badische Aniline- und Soda-Fabrik. Verfahren zur Darstellung von künstlichem Indigo. German Pat..

[122] Curtius T., Bollenbach H.F. (1907). Die Einwirkung von Hydrazinhydrat auf Nitroverbindungen. III. Abhandlung. Über die Einwirkung von Hydrazinhydrat auf 2,4-Dinitrobenzoesäure. J. Prakt. Chem..

[123] (1908). Farbwerke vorm. Meister Lucius & Brüning. Verfahren zur Darstellung von 4-Amino-2-nitrobenzoesäure. German Pat..

[124] Ettinger L., Friedländer P. (1912). Über *N*-Methyl-Derivate des Indigoblaus. Ber. Deutsch. Chem. Ges..

[125] Robertson A., Waters R.B. (1931). Synthesis of glucosides. Part VII. The synthesis of 6-bromoindican. J. Chem. Soc..

[126] Dunn G.E., Prysiazniuk R. (1961). Rates of decarboxylation of substituted anthranilic acids in nitrobenzene solution. Can. J. Chem..

[127] Leggate P., Dunn G.E. (1965). Application of the extended Hammett relationship to the ionization constants of substituted anthranilic acids. Can. J. Chem..

[128] Boojamra C.G., Burow K.M., Thompson L.A., Ellman J.A. (1997). Solid-phase synthesis of 1,4-benzodiazepine-2,5-diones. Library preparation and demonstration of synthesis generality. J. Org. Chem..

[129] Zoppetti G., Oreste P., Cipolletti G., Tamerlani G. (1999). Indole derivatives suitable to be used as chromogenic compounds. PCT Int. Appl..

[130] Holt S.J., Sadler P.W. (1958). Studies in enyme chemistry. II. Synthesis of indigogenic substrates for esterases. Proc. Roy. Soc. London Ser. B. Biol. Sci..

[131] Clark R.J.H., Cooksey C.J. (1997). Bromoindirubins: the synthesis and properties of minor components of Tyrian purple and the composition of the colorant from *Nucella lapillus*. J. Soc. Dyers Color..

[132] Cooksey C.J. (2000).

[133] Waldmann H. (1930). 4-Brom-phthalsäure-anhydrid und Derivate. J. Prakt. Chem..

[134] Badische Aniline- und Soda-Fabrik (1891). Verfahren zur Darstellung von Anthranilsäure. German Pat..

[135] Sabourin E.T., Onopchenko A. (1983). A convenient synthesis of 4-ethynylphthalic anhydride via 2-methyl-3-butyn-2-ol. J. Org. Chem..

[136] Hinde N.J., Hall C.D. (1998). Kinetics and mechanism of the formation of mono- and di-phthalate estes catalysed by titanium and tin alkoxides. J. Chem. Soc. Perkin Trans. 2.

[137] Vainberg O., Shorr L.M. (1991). Bis(4-halo-phthalic acid) quarter salt, process for their preparation and their use. US Pat..

[138] Oren J. (1999). Purification of 4-bromo-phthalic anhydride (4-BPAn). Israel Pat..

[139] Ioffe D., Oren J. (2000). 4-BPA anhydride - a versatile intermediate. Specialty Chem..

[140] Moore T.S., Marrack M.T., Proud A.K. (1921). The application of Hofmann's reaction to substituted phthalimides. J. Chem. Soc..

[141] Milner D.J. (1985). An improved procedure for the synthesis of 3-fluoroanthranilic acid. Synth. Commun..

[142] Schmand H., Kellermeier B., Bartels G., Schmidt H.-J. (1998). Process for the preparation of haloanthranilic acids. US Pat..

[143] Ullmann F. (1903). Ueber eine neue Bildungsweise von Diphenylaminderivaten. Ber. Deutsch. Chem. Ges..

[144] Ullmann F. (1904). Ueber eine neue Darstellungsweise von Phenyläthersalicylsäure. Ber. Deutsch. Chem. Ges..

[145] Wooster C.B. (1963). Ammonolysis. Kirk-Othmer Encyclopedia of Chemical Technology.

[146] Badische Anilin- und Soda-Fabrik (1912). Verfahren zur Darstellung von Chlorsubstitutionsprodukten der Anthranilsäure. German Pat..

[147] Claus A., Weil A. (1892). Zur Kenntniss der Dibrombenzoësäuren. Justus Liebigs Ann. Chem..

[148] Meyer V., Sudborough J.J. (1894). Das Gesetz der Esterbildung aromatischer Säuren. Ber. Deutsch. Chem. Ges..

[149] Sudborough J.J. (1895). Diortho-substituted benzoic acids. II. Hydrolysis of aromatic nitriles and acid-amides. J. Chem. Soc..

[150] Gomberg M., Cone L.H. (1909). Ueber Triphenylmethyl: XVIII. Mittheilung: Zur Kenntniss der Chinocarboniumsalze. Justus Liebigs Ann. Chem..

[151] Montagne P.J., van Charante J.M. (1912). De l’action d’une solution alcoholique de potasse caustique sur les cétones (Deuxième mémoire). II. Rec. Trav. Chim. Pays-Bas Belg..

[152] Olivier S.C.J. (1929). Sur la saponification des nitriles d'après la méthode à l'acide orthophosphorique. Rec. Trav. Chim. Pays-Bas Belg..

[153] Doering W., von E., Sayigh A.A.-R. (1954). On the reported rearrangement of 2,4,7-tribromotropone to 3,5-dibromobenzamide. J. Am. Chem. Soc..

[154] Blackhall A., Brydon D.L., Javaid K., Sagar A.J.G., Smith D.M. (1984). Substitution reactions of phenylated aza-heterocycles. Part 2. Bromination of some 2,5-diaryl-1,3,4-oxadiazoles. J. Chem. Res..

[155] Corbett T.H., Grossman C.S., Lobb K.L., Shih C., Hipskind P.A., Lin H.-S. (2002). Preparation of benzoylsulfonamide and sulfonylbenzamide angiogenesis inhibitors for use as antitumor agents. PCT Int. Appl..

[156] De Dios A., Grossman C.S., Hipskind P.A., Lin H.-S., Lobb K.L., Lopez de Uralde Garmendia B., Lopez J.E., Mader M.M., Richett M.E., Shih C. (2003). Preparation of thiophene- and thiazolesulfonamides as antineoplastic agents. PCT Int. Appl..

[157] Nevile R.H.C., Winther A. (1880). Ueber die Constitution der Produkte der Einwirkung von Brom und Salpetersäure auf aromatische Amidokörper und die sechs isomeren Dibromtoluole. Ber. Deutsch. Chem. Ges..

[158] Miller A.K. (1892). Studies on the interaction of bromine and toluene. Preparation and properties of orthobromotoluene and parabromotoluene, and of the dibromotoluenes derivable therefrom. Ortho- and para-bromotoluenesulphonic acids. J. Chem. Soc..

[159] Cohen J.B., Zortman I.H. (1906). The relation of position isomerism to optical activity. V. The rotation of the menthyl esters of the isomeric dibromobenzoic acids. J. Chem. Soc..

[160] Cohen J.B., Dutt P.K. (1914). The progressive bromination of toluene. J. Chem. Soc..

[161] Olivier S.C.J. (1925). Sur l'introduction de deux atomes de brome dans l’acétométatoluidide. Rec. Trav. Chim. Pays-Bas Belg..

[162] Olivier S.C.J. (1926). L'hydrolyse des chlorures de benzyle substitutes et la théorie de l’empéchment stérique. Rec. Trav. Chim. Pays-Bas Belg..

[163] Motsarev G.V., Yakubovich A.Y. (1957). Halogenation of aromatic silanes. IV. Preparation and properties of chloro and bromo derivatives of p-tolyltrichlorosilane. J. Gen. Chem. USSR.

[164] Ledóchowski A., Ledóchowski Z. (1959). Research of tumour inhibiting substances. II. The synthesis of some derivatives of 1-bromo-7-methoxy-9-aminoacridine. Rocz. Chem..

[165] Yamanari K., Ishimura K. (1970). Pyrolysis of potassium 2,4-dibromobenzoate. Seikei Dagaku Kogakubu Kogaku Hokoku.

[166] Standard Oil Company (1971). Production of nuclear brominated benzene carboxylic acids. Brit. Pat.

[167] Tashiro M., Nakayama K. (1984). Preparation of bromobenzoic acids from the corresponding bromotoluenes via the Krohnke method. Org. Prep. Proc. Int..

[168] Nippon Kayaku Co., Ltd. (1981). 2-Halobenzonitrile derivatives. Japanese Pat..

[169] Hussey A.S., Dyer J.R. (1951). The monomagnesium derivatives of dibromotoluenes. J. Am. Chem. Soc..

[170] Moodie R.B., Schofield K., Weston J.B. (1976). Electrphilic aromatic substitution. Part XV. The kinetics, mechanism, and products of nitrodebromination in sulphuric acid. J. Chem. Soc. Perkin 2.

[171] Areschka A., Bruylants A. (1958). Méthode spectrographique de detection des méthylènes actifs et synthèse de quelques dérivés styryliques et stilbéniques. VII. – Les derives bromes du nitrile phénylacétique (I). Bull. Soc. Chim. France.

[172] Pajeau R. (1960). Action du brome en presence d’éthers-oxydes. Bull. Soc. Chim. France.

[173] Tapolczay D.J., Spinney M.A. (1989). Insecticides. US Pat..

[174] Venkatachalapathy C., Pitchumani K. (1997). Selectivity in bromination of alkylbenzenes in the presences of montmorillonite clay. Tetrahedron.

[175] Rozen S., Brand M., Lidor R. (1988). Aromatic bromination using BrF with no Friedel-Crafts catalyst. J. Org. Chem..

[176] Bromine Compounds Ltd. (1992). Process for the ring bromination of benzenoid compounds. Israel Pat..

[177] Kanti R.B., Nargund K.S. (1956). Dibromoacetophenones. J. Karnatak Univ..

[178] Troyanov I.A., Dibinskaya A.A. (1963). On acetylation of *p*-dibromobenzene. Ukr. Khim. Zh..

[179] Mathieson D.W., Newberry G. (1949). Contributions to the chemistry of synthetic antimalarials. Part VIII. Aromatic carbinolamines. J. Chem. Soc..

[180] Śledziński B., Cieślak L. (1973). Synthesis of halophenacylidine halides. Rocz. Chem..

[181] Gibson C.S., Levin B. (1931). Benzaldehyde-*p*-arsonic acid, arsonic acids of acylphenylketones and their derivatives. Chemotherapeutic examination of these and other arsenic acids. J. Chem. Soc..

[182] Koton M.M., Kiseleva T.M., Florinskii F.S. (1953). Synthesis and polymerization of nuclearly substituted dibromostyrenes. Zh. Prikl. Khim..

[183] Kuliev A.M., Mirzoev B.M., Hoe N., Farzaliev V.M. (1971). Synthesis of 2,5-dialkylstyrenes and 2,5-dihalogenostyrenes and their condensation with formaldehyde. Zh. Org. Khim..

[184] Polívka Z., Jílek J., Holubek J., Svátek E., Dlabač, Valchář M., Protiva M. (1984). Noncataleptic neuroleptic agents: 2-Halogeno-8-isopropyl-10-piperazino-10,11-dihydrodibenzo[*b*,*f*]thiepins. Coll. Czech. Chem. Commun..

[185] Hellwinkel D., Bohner S. (1987). Dibenzocycloocten-, Dibenzochalcocin- und Diarenochalconindione. Chem. Ber..

[186] Leroy A.J. (1887). Sur les benzines bromées. Bull. Soc. Chim. France.

[187] Holleman A.F., van der Linden T. (1911). Etudes sur la formation simultanée de produits de substitution isomers du benzene. Recherches quantitatives sur l’introduction d’un deuxième atome d’halogène dans les benzenes monohalogénés. Rec. Trav. Chim. Pays-Bas.

[188] Copisarov M., Long C.N.H. (1921). The Friedel-Crafts’ reaction. Part II. Migration of the halogen atoms in the benzene nucleus. J. Chem. Soc..

[189] Olah G.A., Tolgyesi W.S., Dear R.E.A. (1962). Friedel-Crafts isomerization. III. Aluminum bromide-catalyzed isomerization of bromofluorobenzenes, bromochlorobenzenes, and dibromobenzenes. J. Org. Chem..

[190] Shell International Research (1962). A process for the isomerization of para dibromobenzene to meta dibromobenzene. Brit. Pat.

[191] Sax K.J. (1962). Halogenation process. US Pat..

[192] Tolgyesi W.S. (1967). Meta-substituted halobenzenes. US Pat..

[193] Reith H., Schimming R., Krause J. (1972). Verfahren zur Hestellung von an m-Dihalogenbenzolreichen Gemischen, vorzugsweise als Ausgangsprodukt für die Synthese von Polyphenyläther-Scmierstoff.

[194] Perrier G. (1900). Ueber die Rolle des Aluminiumchlorids bei der Friedel-Crafts’schen Reaction. Ber. Deutsch. Chem. Ges..

[195] Perrier G. (1904). A propos d’une note de M. C. Graebe sur un benzoylacénaphthène. Réclamation de priorité. Bull. Soc. Chim. France.

[196] VanArendonk A.M., Cupery M.E. (1931). The reaction of acetophenone derivatives with sodium hypochlorite. J. Am. Chem. Soc..

[197] Fieser L.F., Holmes H.L., Newman M.S. (1936). The oxidation of methyl *α*- and *β*-naphthyl ketones. J. Am. Chem. Soc..

[198] Newman M.S., Holmes H.L. (1943). β-Naphthoic acid. Org. Synth. Coll..

[199] Farrar M.W., Levine R. (1949). The oxidation of certain ketones to acids by alkaline hypochlorite solution. J. Am. Chem. Soc..

[200] Perkins R. (1984). Synthesis of benzoic acid using household bleach. J. Chem. Ed..

[201] Flemming W., Speer W. (1935). Catalytic oxidation of ketones. US Pat..

[202] Stubbs J.J., Senseman C.E. (1936). 4-Chloroacetophenone: Catalytic oxidation in the liquid phase. Ind. Eng. Chem..

[203] Slotterbeck O.C. (1941). Catalytic oxidation of ketones. U. S. Pat..

[204] Amend W.J. (1943). Catalytic oxidation. U. S. Pat..

[205] Fumagalli C., Minisci F., Pirola R. (2001). Process for preparation of aromatic carboxylic acids. PCT Int. Appl..

[206] Hay A.S., Blanchard H.S. (1965). Autoxidation reactions catalyzed by cobalt acetate bromide. Can. J. Chem..

[207] Tmenov D.N., Lysukho T.V., Shcherbina F.F., Belous A.I., Kornyushkina T.G. (1977). Liquid-phase catalytic oxidation of chlorotoluenes. J. Appl. Chem. USSR.

[208] Effenberger R., Michman M. (1983). Production of derivatives of benzoic acid. Israel Pat..

[209] Shcherbina F.F. (1989). Preparation of o-chlorobenzoic acid by liquid-phase catalytic oxidation of o-chlorotoluene. J. Appl. Chem. USSR.

[210] Yoshino Y., Hayashi Y., Iwahama T., Sakaguchi S., Ishii Y. (1997). Catalytuc oxidation of alkylbenzenes with molecular oxygen under normal pressure and temperature by *N*-hydroxyphthalimide combined with Co(OAc)_2_. J. Org. Chem..

[211] Hirai N., Sawatari N., Nakamura N., Sagaguchi S., Ishii Y. (2003). Oxidation of substituted toluenes with molecular oxygen in the presence of *N*,*N′*,*N″*-trihydroxyisocyanuric acid as a key catalyst. J. Org. Chem..

[212] Farbwerke vorm. Meister Lucius & Brüning (1901). Verfahren zur Darstellung von Indigo und seinen stickstoffalkylirten Derivaten. German Pat..

[213] Farbwerke vorm. Meister Lucius & Brüning (1901). Verfahren zur Darstellung von Phenylglycin-o-carbonsäure. German Pat..

[214] Farbwerke vorm. Meister Lucius & Brüning (1903). Verfahren zur Darstellung von. Phenylglycin-o-carbonsäure. German Pat..

[215] Farbwerke vorm. Meister Lucius & Brüning (1903). Verfahren zur Darstellung von Phenylglycin-o-carbonsäure. German Pat..

[216] Krause S., Neumann-Grimm D., Papenfuhs T. (1998). Process for the preparation of N-(2-carboxy-5-chloro-phenyl)glycine. U. S. Pat..

[217] Papenfuhs T., Pfirmann R., Krause S., Neumann-Grimm D. (1999). Process for preparing N-carboxymethylene-4-chloroanthranilic acid and its dialkyl esters. US Pat..

[218] Kamiya T., Takehara H., Inamoto Y., Taniguchi A., Masumoto M. (2001). Process for the preparation of 3-dihalobenzyl-2,4-quinazolinedione derivatives. U.S. Pat.

[219] Ledóchowski Z., Ledóchowski A., Bosowski E., Wysocka B., Kikmunter A., Morawski B., Gawle K., Wypych H. (1960). Research of tumour inhibiting substances. IV. The synthesis of some derivatives of 9-(4-dimethyl-aminobutylamino)-acridine. Rocz. Chem..

[220] Butlin R.J., Glarvey D. (1992). Preparation of 9,10-dihydroacridine-9-one derivatives.

[221] Magidson O.Y., Grigorovskii A.M., Gal'perin E.P. (1938). Acridine compounds as a source for remedies. IV. The relation between antimalarial effect and changing substituents in position 2 and 6 as well as the amine in the side chain. Zhur. Obshch. Khim..

[222] Cohen J.B., Raper H.S. (1904). The relation of position isomerism to optical activity. II. The rotation of the menthyl esters of the isomeric chlorobromobenzoic acids. J. Chem. Soc..

[223] Cohen. J.B., Smithells C.J. (1914). The chlorination and bromination of substituted toluenes. J. Chem. Soc..

[224] Motsarev G.V., Yakubovich A.Y. (1958). Halogenation of aromatic silanes. VI. Preparation and properties of p-(trichloromethyl)phenyltrichlorosilane. J. Gen. Chem. USSR.

[225] Bergmann E.D., Barshai R. (1959). 2,3-Dichloroindone as a dienophilic compound. J. Am. Chem. Soc..

[226] Guo Z., Liao R., Yang H., Pu J., Wang Y., Gong X., Yang Y., Li Z. (1993). Synthesis of new sedative and antiepileptic 2-chloro-4-bromo-α-methylcinnamic acid. Huaxi Yaoxue Zazhi.

[227] Stock L.M., Baker F.W. (1962). Rates and isomer distributions in the non-catalytic chlorination of the halobenzenes and certain halotoluenes in aqueous acetic acid. Partial rate factors for the halogenation of the halobenzenes. J. Am. Chem. Soc..

[228] Vainshtein F.M., Tomilenko E.I., Shilov E.A. (1969). Investigation of the exchange of bromine and chlorine in halosubstituted benzenecarboxykic acids under the action of ammonia in the presence of copper (I). Kinet. Catal..

[229] Chumak V.T., Shein S.M. (1985). General basic and coordination catalysis in reactions of haloaromatic compounds with ammonia in the presence of copper (I) compounds. Kinet. Catal..

[230] Grandmougin E., Seyder P., Über Indigo V. (1914). Über halogenierte Indigo und Derivate. Ber. Deutsch. Chem. Ges..

[231] Schwarz J. (1905). Darstellung von 4-Dinitroindigo. Monatsh. Chem..

[232] Sadler P. W. (1956). Absorption spectra of indigoid dyes. J. Org. Chem..

[233] Nölting E., Collin A. (1884). Ueber das Nitroorthotoluidin Schmelzpunkt 107º und einige seiner Abkömmlinge. Ber. Deutsch. Chem. Ges..

[234] Wheeler H.L., Barnes B. (1898). On the silver salt of 4-nitro-2-aminobenzoic acid and its behavior with alkyl and acyl halides. Am. Chem. J..

[235] Ullmann F., Uzbachian J.B. (1903). Ueber die Verwendung von Permanganaten als Oxydationsmittel. Ber. Deutsch. Chem. Ges..

[236] Friedländer P., Cohn P. (1902). Ubero *p*-Dinitrobenzaldehyd. Monatsh. Chem..

[237] Holt S.J., Petrow V. (1947). Carbazoles, carbolines, and related compounds. Part I. Quindoline derivatives. J. Chem. Soc..

[238] Majima R., Kotake M. (1930). Synthetische Versuche in der Indol-Gruppe, VII: Über Nitrieren und Bromieren des β-Indol-carbonsäure-esters und eine neue Synthese des Farbstoffs des antiken Purpurs. Ber. Deutsch. Chem. Ges..

[239] Oddo B. (1911). Synthesis in the indole group. I. Alkylindoles. Gazz. Chim. Ital..

[240] Majima R., Kotake M. (1922). Synthetische Versuche in der Indolgruppe, II.: Über den Einfluβ von Lösungsmitteln auf die Grignardische Reaktion. Ber. Deutsch. Chem. Ges..

[241] Oddo B., Sessa L. (1911). Synthesis in the indole group. I. Alkyl indolyl ketones and indolic Acids. Gazz. Chim. Ital..

[242] Shirley D.A., Roussel P. (1953). Metalation of indole, N-methylindole and N-phenylindole with *n*-butyllithium. J. Am. Chem. Soc..

[243] Kašpárek S., Heacock R.A. (1967). The chemistry of the indole Grignard reagent. II. The reaction of indole magnesium iodide with carbon dioxide. Can. J. Chem..

[244] Leggetter B.E., Brown R.K. (1960). The structure of monobrominated ethyl indole-3-carboxylate and the preparation of 7-bromoindole. Can. J. Chem..

[245] Tanoue Y., Terada A., Sakata K., Hashimoto M., Morishita S.-I., Hamada M., Kai N., Nagai T. (2001). A facile synthesis of Trian purple based on a biosynthetic pathway. Fisheries Sci..

[246] Pappalardo G., Vitali T. (1958). Indoles. IV. Ultraviolet absorption spectra of haloindoles and halo-2-indolecarboxylic acids and ester. Gazz. Chim. Ital..

[247] Piers E., Brown R.K. (1960). The decarboxylation of ring-substituted indole-2(and 3)-carboxylic acids. Can. J. Chem..

[248] Da Settimo A., Saettone M.F., Nannipieri E., Barili P. (1967). La bromurazione di alcuni derivati indolici. Gazz. Chim. Ital..

[249] Anderson J.A., Kohler R.D. (1974). Decarboxylierung von Bromoindol-2-carbonsäuren. Synthesis.

[250] Schreier E. (1976). Radiolabelled peptide ergot alkaloids. Helv. Chim. Acta.

[251] Schmidt U. (1982). Total synthesis of tetraacetyl clionamide. Tetrahedron Lett..

[252] Schmidt U., Lieberknecht A., Griesser H., Bökens H. (1985). Totalsynthese und biomimetische Bildung von Clionamid-Derivaten. Justus Liebigs Ann. Chem..

[253] Suzuki H., Gyuotoku H., Yokoo H., Shinba M., Sato Y., Yamada H., Murakami Y. (2000). Unexpected formation of quinoline derivatives in Reissert indole synthesis. Synlett.

[254] Jones G.B., Chapman B.J. (1993). Decarboxylation of indole-2-carboxylic acids: Improved procedures. J. Org. Chem..

[255] Dellar G., Djura P., Sargent M.V. (1981). Structure and synthesis of a new bromoindole from a marine sponge. J. Chem. Soc Perkin 1.

[256] Moyer M.P., Shiurba J.F., Rapoport H. (1986). Metal-halogen exchange of bromoindoles. A route to substituted indoles. J. Org. Chem..

[257] Ayer W.A., Craw P.A., Ma Y., Miao S. (1992). Synthesis of camalexin and related phytoalexins. Tetrahedron.

[258] Sinclair P.J., Goulet M. (1992). O-heteroaryl, O-alkylheteroaryl, O-alkenylheteroaryl and O-alkynylheteroaryl macrolides. CA Pat..

[259] Rasmussen T., Jensen J., Anthoni U., Christophersen C., Nielsen P.H. (1993). Structure and synthesis of bromoindoles from the marine sponge *Pseudosuberites* hyalinus. J. Nat. Prod..

[260] Carrera G.M., Sheppard G.S. (1994). Synthesis of 6- and 7-arylindoles *via* palladium-catalyzed cross-coupling of 6- and 7-bromoindole with arylboronic acids. Synlett.

[261] Jiang B., Smallheer J.M., Amaral-Ly C., Wuonola M.A. (1994). Total synthesis of (±)-dragmacidin: A cytotoxic bis(indole)alkaloid of marine origin. J. Org. Chem..

[262] Dombroski M., Eggler J.F. (1998). Sulfonyl urea derivatives and their use in the control of interleukin-1 activity. PCT Int. Appl..

[263] Schumacher R.W., Davidson B.S. (1999). Synthesis of didemnolines A-D, N9-substituted β-carboline alkaloids from the marine ascidian *Didemnum* sp.. Tetrahedron.

[264] Orme M.W., Sawyer J.S., Daugan A.C.-M. (2001). Preparation of cyclic guanosine monophosphate specific phosphodiesterase inhibiting heterocyclylpyrazinopyridoindolediones for treatment of cardiovascular disorders and erectile disfunction. PCT Int. Appl..

[265] Konda-Yamada Y., Okada C., Yoshida K., Umeda Y., Arima S., Sato N., Kai T., Takayanagi H., Harigaya Y. (2002). Convenient synthesis of 7′- and 6′-bromo-D-tryptophan and their derivatives by enzymatic optical resolution using D-aminoacylase. Tetrahedron.

[266] Chen Z., Cohen M.P., Fisher M.J., Giethlen B., Gillig J.R., McCowan J.R., Miller S.C., Schaus J.M. (2002). N-(2-arylethyl)benzylamines as antagonists of the 5-HT_6_ receptor. PCT Int. Appl..

[267] Toste F.D., Still I.W.J. (1995). Sonication and aluminum amalgam in the Leimgruber-Batcho reaction: An improved preparation of 6-aminoindole. Org. Prep. Proc. Int..

[268] Miyake Y., Kikugawa Y. (1983). Synthesis of 6-methoxyindoles and indolines. Regioselective C-6 bromination of indolines and subsequent nucleophilic substitution with a methoxyl group. J. Heterocycl. Chem..

[269] Kawase M., Miyaka Y., Kikugawa Y. (1984). Dehydrogenation of indolines to indoles *via* azasulphonium salts or *N*-chloramines. J. Chem. Soc. Perkin 1.

[270] Inoue Y., Yamamoto Y., Suzuki H., Takaki U., Corberán. V.C., Bellón S.V. (1994). Novel one-pot synthesis of indigo from indole and organic hydroperoxide. New Developments in Selective Oxidation.

[271] Wolk J., Frimer A.A. (2010). A simple, low cost, safe, high yield synthesis of Tyrian purple. Molecules.

[272] Fieser L.F., Fieser M. (1967). Reagents for Organic Synthesis.

[273] Goldberg A.A. (1952). Hydrolysis of substituted *o*-chlorobenzoic acids. The mechanism of the reaction between *o*-halogenobenzoic acids and nucleophilic reagents. J. Chem. Soc..

[274] Pellón R.F., Carrasco R., Millián V., Rodés L. (1995). Use of pyridine as cocatalyst for the synthesis of 2-carboxy substituted diphenylethers by Ullmann-Goldberg condensation. Synth. Commun..

[275] Pellón R.F., Mamposo T., Carrasco V., Rodés L. (1996). Use of pyridine as cocatalyst for the synthesis of N-phenylanthranilic acids. Synth. Commun..

